# Domestication Explains Two-Thirds of Differential-Gene-Expression Variance between Domestic and Wild Animals; The Remaining One-Third Reflects Intraspecific and Interspecific Variation

**DOI:** 10.3390/ani11092667

**Published:** 2021-09-10

**Authors:** Irina Chadaeva, Petr Ponomarenko, Rimma Kozhemyakina, Valentin Suslov, Anton Bogomolov, Natalya Klimova, Svetlana Shikhevich, Ludmila Savinkova, Dmitry Oshchepkov, Nikolay A. Kolchanov, Arcady Markel, Mikhail Ponomarenko

**Affiliations:** Institute of Cytology and Genetics, Siberian Branch of Russian Academy of Sciences, 630090 Novosibirsk, Russia; ichadaeva@bionet.nsc.ru (I.C.); pon.petr@gmail.com (P.P.); korimma@gmail.com (R.K.); valya@bionet.nsc.ru (V.S.); mantis_anton@bionet.nsc.ru (A.B.); klimova@bionet.nsc.ru (N.K.); shikhsvt@bionet.nsc.ru (S.S.); lksav@bionet.nsc.ru (L.S.); diman@bionet.nsc.ru (D.O.); kol@bionet.nsc.ru (N.A.K.); markel@bionet.nsc.ru (A.M.)

**Keywords:** *Rattus norvegicus*, domestication, RNA-Seq, qPCR, differentially expressed gene, principal component analysis, intraspecific variety, interspecific variation

## Abstract

**Simple Summary:**

Before genomes were sequenced, zoologists had discovered destabilizing selection as a general pattern of animal domestication that in foxes and minks had yielded fur colors never seen in the wild. Today, known genomes of humans and domestic and wild animals arouse interest in a common whole-genome pattern of animal domestication that may at least explain differences between the effects of natural and artificial environments on organisms. Accordingly, here we identified genes differing in expression between tame and aggressive rats (a laboratory domestication model) to compare them with all such known changes of genes’ activity (available in databases) in other domestic versus wild animals (e.g., dogs versus wolves). The results show that the majority of these genes changed their expression similarly among all the domestic versus wild animals studied, i.e., explained two-thirds of the variance, while the remaining one-third reflected animal intraspecific and interspecific variation, just as the gold standard of variation in nature. Accordingly, here we found that the artificial environment of domestic animals alters activities of genes in the same direction as that seen in corresponding human genes during human diseases, whereas the natural environment maintains a normal gene expression pattern in wild animals (matching human health).

**Abstract:**

Belyaev’s concept of destabilizing selection during domestication was a major achievement in the XX century. Its practical value has been realized in commercial colors of the domesticated fox that never occur in the wild and has been confirmed in a wide variety of pet breeds. Many human disease models involving animals allow to test drugs before human testing. Perhaps this is why investigators doing transcriptomic profiling of domestic versus wild animals have searched for breed-specific patterns. Here we sequenced hypothalamic transcriptomes of tame and aggressive rats, identified their differentially expressed genes (DEGs), and, for the first time, applied principal component analysis to compare them with all the known DEGs of domestic versus wild animals that we could find. Two principal components, PC1 and PC2, respectively explained 67% and 33% of differential-gene-expression variance (hereinafter: log_2_ value) between domestic and wild animals. PC1 corresponded to multiple orthologous DEGs supported by homologs; these DEGs kept the log_2_ value sign from species to species and from tissue to tissue (i.e., a common domestication pattern). PC2 represented stand-alone homologous DEG pairs reversing the log_2_ value sign from one species to another and from tissue to tissue (i.e., representing intraspecific and interspecific variation).

## 1. Introduction

One of the key achievements of genetics in the XX century is Belyaev’s concept of destabilizing selection during animal domestication as the most common pattern of domestication, namely: “In a genetic and biochemical sense, what may be selected for are changes in the regulation of genes —that is, in the timing and the amount of gene expression rather than changes in individual structural genes. Selection having such an effect is called by me destabilizing selection. The selection becomes destabilizing when it affects, directly or indirectly, the systems of neuroendocrine control of ontogenesis. This seems always to be the case when some new stressful factors appear in the environment, or when stresses usual for the species increase in strength.” [1]. This breakthrough summed up the results of long-term unique experiments on both mink (e.g., [2]) and fox (e.g., [3]) domestication as well as on mice as a laboratory model of human diseases (e.g., [4]). Among these experiments was our study on how emotionally mice respond to stress [5]. The practical value of Belyaev’s concept has been realized in a huge collection of commercial fur colors—e.g., “Amber-gold pastel,” “Ashen,” “Beige,” “Black crystal,” “Cobalt,” “Ermine-like,” “Peach,” ““Pearl,” Platinum,” “Purple,” “Silver sable-like,” “Steel-blue,” and “Straw,”—rarely or never seen in the wild [6]. The concept’s fundamental importance has found confirmation in a large variety of breeds of dogs [7], cats [8], pigs [9], cows [10], horses [11], sheep [12], goats [13], chickens [14], ducks [15], geese [16], and other domestic animals [17] as well as in artificial shelters and additional feeding for wild animals in wildlife for saving them [18]. On the basis of Belyaev’s concept [1], a laboratory model of animal domestication by humans has been created using outbred lines of tame and aggressive rats artificially bred [19] for performance on a standard glove test [20]. Moreover, within translational biomedicine [21], a lot of the human disease models involving domestic and laboratory animals, including transgenic ones [22], are actively researched to eventually conduct preclinical trials of drugs intended for human treatments. Perhaps that is why the majority of transcriptome-profiling studies on domestic versus wild animals are still focused on the search for the species specificity of differentially expressed genes (DEGs) that has practical value [23,24,25,26,27,28,29] rather than for common patterns of domestication. An exception from the mainstream in genome-wide studies on animals is a comparison of the human variome with differences between domestic and wild animals, where the researchers have generalized their results by means of the new concept of self-domestication syndrome, the symptoms of which include autism spectrum disorders [30], although the idea of human self-domestication is still debatable [31].

Among the studies fitting Belyaev’s concept of destabilizing selection during domestication [1], in our previous works, we have measured the murine emotional response under stress [5]. Next, our genome-wide study of the CpG-islands within gene promoters in humans, chimpanzees, gorillas, and orangutans identified the evolution of brain active promoters in the human lineage toward their increased epigenetic regulation plasticity [32]. Moreover, we have conducted a genome-wide study on single-nucleotide polymorphisms (SNP) within gene promoters of the human nervous system and found that natural selection equally supports propensities to domination and subordination, which must be inherited from parent to offspring; we proved this idea using F1 hybrid mice [33]. Additionally, by quantitative polymerase chain reaction (qPCR), we have identified seven hypothalamic DEGs of tame versus aggressive rats [34] as the above-mentioned laboratory model of animal domestication [19,20]. Finally, applying the factor analysis [35] to these seven rat DEGs, we for the first time observed that artificial selection for behavior, adaptation to laboratory living conditions, and intraspecific variety equally well explain the differential-gene-expression variance of tame versus aggressive rats [36]. That is why the present work generalizes our above-mentioned qPCR-based findings at the whole-genome scale. Biomedical applications of the results are discussed at the end of the paper.

## 2. Materials and Methods

### 2.1. Animals

The study was performed on adult male gray rats (*Rattus norvegicus*) selectively bred for over 90 generations for either aggressive or domesticated behavior (as two outbred lines) under standard conditions of the Conventional Animal Facility of the Institute of Cytology and Genetics (ICG), the Siberian Branch of the Russian Academy of Sciences (SB RAS; Novosibirsk, Russia), as described elsewhere [19,20,37]. The total number of rats was 22 (11 aggressive and 11 domesticated), each weighing 250–270 g and 4 months old, all from different unrelated litters. All the rats were decapitated. Following a handbook [38], we extracted samples of the hypothalamus, which were then flash-frozen in liquid nitrogen and stored at −70 °C until use. We focused on the hypothalamus as a universal brain region most frequently used in studies on aggressiveness of female and male animals of all ages (for review, see e.g., [39]). Every effort was made to minimize the number of animals studied and their suffering.

### 2.2. RNA-Seq

For RNA isolation, approximately 100 mg of tissue was used. Total RNA from hypothalamus samples of tame (*n* = 3) and aggressive (*n* = 3) rats for sequencing was isolated using the TRIzol™ reagent (Invitrogen, Carlsbad, CA, USA, cat. #15596018) according to the manufacturer’s protocol. By means of the mirVana™ Kit (miRNA Isolation Kit without phenol, Thermo Fisher Scientific, AB-AM1561), the RNA was separated. The total RNA after separation on mirVana columns was analyzed quantitatively by means of an Invitrogen Qubit™ 2.0 fluorometer (Invitrogen/Life Technologies, Carlsbad, CA, USA).

The quality of total RNA was assessed with the RNA Nano Kit on an Agilent Bioanalyzer 2100 bioanalyzer (Agilent, Santa-Clara, CA, USA). Samples with RNA Integrity Numbers (RINs) of 7.0 to 8.0 were chosen for further analysis. To obtain a highly purified mRNA fraction, the Dynabeads mRNA Purification Kit (Invitrogen) was employed in accordance with the manufacturer’s protocol. For purification, 5 μg of the RNA fraction depleted of small RNAs was used. The absence of significant degradation of RNA and the presence of rRNA impurities at less than 20% were monitored on a bioanalyzer with the RNA Pico Kit. To create barcoded RNA-Seq libraries, 15–30 ng of mRNA was used; the ScriptSeq™ v2 RNA-Seq Library Preparation Kit (epicenter^®^, Madison, WI, USA) was utilized according to the manufacturer’s protocol. When amplifying the libraries, we carried out 13 polymerase chain reaction (PCR) cycles; the final purification was performed on AMPure XP magnetic beads. The quality of the obtained libraries and their molarity were checked on a bioanalyzer using the DNA High Sensitivity kit; before loading, the library was diluted 1:10. The molarity of the 24 obtained libraries was in the range of 10,000–47,000 pmol/L. The libraries were pooled in equimolar amounts, adjusted to a concentration of 2 nM, and transferred to the Multi-Access Center of Genomic Research (ICG SB RAS, Novosibirsk, Russia) for sequencing on an Illumina NextSeq 550 instrument in a NextSeq^®^ 500/550 High Output Kit v2 cassette (75 cycles) under the assumption of a direct read of 75 nucleotides, with at least 40 million reads. The total volume of sequencing for the 6 libraries was 219,086,104 reads, which were finally deposited in the NCBA SRA database (ID = PRJNA668014) [40].

### 2.3. Mapping of RNA Sequences to the R. norvegicus Reference Genome

The quality of the obtained raw Fastq files was checked and analyzed with FastQC. To improve the quality of the raw reads, we employed the Trimmomatic tool [41] by these procedures: removing a base from either the start or end position if the quality was low, trimming bases by a sliding-window method, and removing any remaining reads that are <36 bases long. The trimmed reads were aligned to the annotated *R. norvegicus* genome retrieved from the UCSC database (RGSC Rnor_6.0, UCSC version Rn6, July 2014 assembly). Alignment was performed in TopHat2 [42]. The alignments were postprocessed into sorted BAM files by means of SAMTools version 1.4 [43]. Reads were attributed to genes using the htseq-count tool from the “HTSeq” framework version 0.7.2 [44] based on gtf-files with coordinates of genes from Rnor_6.0 and an indexed SAM file. Differential expression analysis was performed in DESeq2 [45] on the IRIS web server, which is publicly available at http://bmbl.sdstate.edu/IRIS/ (accessed on 16 January 2020). Genes were considered differentially expressed if they showed an adjusted *p* value of less than 0.05 to ensure statistical significance [46], as widely accepted in the case of DEGs in domestic vs. wild animals (e.g., [23,25,29]).

### 2.4. qPCR

As independent selective verification of the DEGs identified using the above-described RNA-Seq (see Section 2.2 and Section 2.3), in this work, we for the first time examined the total RNA from only the remaining hypothalamus samples of tame (*n* = 8) and aggressive (*n* = 8) rats, which was intended only for this qPCR control assay. That total RNA was also isolated with TRIzol and then purified on Agencourt RNAClean XP Kit magnetic beads (Beckman, #A63987). The amount of RNA was evaluated on a Qubit™ 2.0 fluorometer (Invitrogen/Life Technologies) with a reagent kit (RNA High Sensitivity, Invitrogen # In = Q32852) according to the manufacturer’s instructions. cDNA was synthesized with Reverse Transcription Kit reagents (Syntol, #OT-1). Oligonucleotide primers for qPCR were designed in PrimerBLAST [47], as shown in Table 1. qPCR was conducted with the EVA Green I Kit following the manufacturer’s instructions. The qPCR was carried out in three technical replicates on LightCycler^®^ 96 (Roche, Basel, Basel-Stadt, Switzerland). The efficiency of the qPCR was determined by means of a series of cDNA dilutions (standards). Four rat genes—*B2m* (β-2-microglobulin), *Hprt1* (hypoxanthine phosphoribosyltransferase 1), *Ppia* (peptidylprolyl isomerase A), and *Rpl30* (ribosomal protein L30)—served as reference genes according to published recommendations [48,49,50,51].

Finally, using these qPCR-based magnitudes together with the standard software Statistica (Statsoft^TM^, Tulsa, OK, USA), we selectively verified the aforementioned RNA-Seq data (see Section 2.2) by both the Mann–Whitney *U* test and Fisher’s Z-test as well as both linear and rank correlations.

### 2.5. DEGs of Domestic Animals versus Their Wild Congeners

In this work, we utilized publicly available independent experimental RNA-Seq datasets from transcriptomes of domestic animals compared with their wild congeners [23,24,25,26,27,28,29]; these data were statistically significant according to Fisher’s Z-test, with corrections for multiple comparisons (P_ADJ_ < 0.05), as published by their authors (see, the PubMed database [52]) and outlined in Table 2.

**Figure 1:**
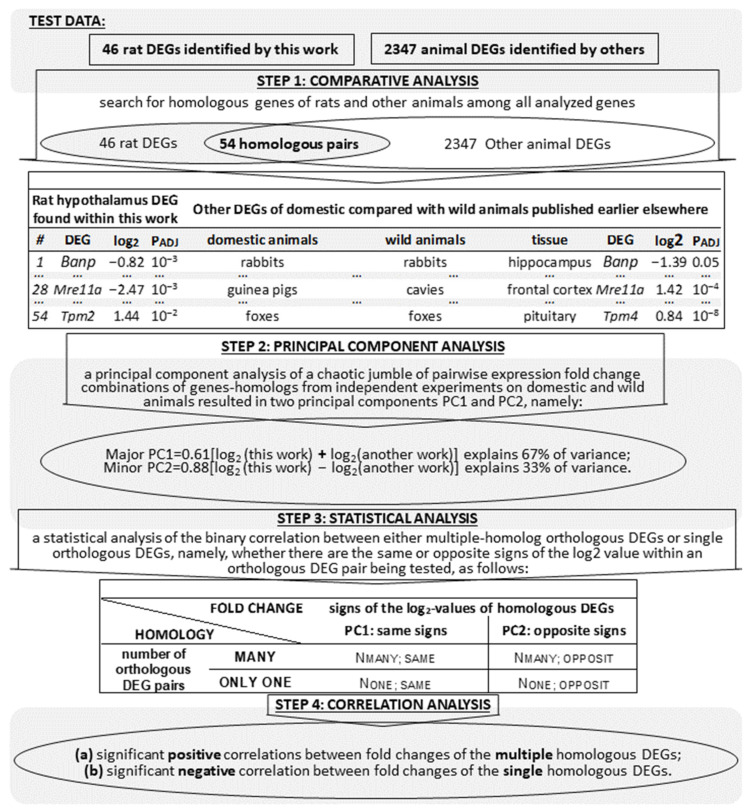
An algorithmic flowchart illustrating the verification of the DEGs found in this study in the hypothalamus of tame versus aggressive rats with respect to their known homologous DEGs in domestic animals compared with their wild congeners, as independently reported by others. Legend: log2, the log_2_-transformed fold change (i.e., the ratio of a domestic-animal gene expression level to that in wild animals); PC1 and PC2: first and second principal components, respectively.

As a result, 2347 DEGs were analyzed, which represented seven tissues of seven domestic animal species and seven of their wild congeners, as readers can see in in the last row of Table 2. Figure 1 represents an algorithmic flowchart detailing the verification of the DEGs found in this work in the hypothalamus of tame versus aggressive rats with respect to their known homologous DEGs in domestic animals versus their wild congeners, as independently reported by others (Table 2), as depicted by the “TEST DATA” area. In brief, we first searched for homologous genes of rats and other animals among all analyzed genes (STEP 1). Next, applying a principal component analysis to these pairwise expression-fold-change combinations of genes-homologs, we found two principal components PC1 and PC2 (STEP 2). Then, we conducted a statistical analysis of the correlation between either multiple-homolog orthologous DEGs or single orthologous DEGs, namely, we tested whether there are the same (PC1) or opposite (PC2) signs of the log_2_-transformed ratio of an expression level of a given gene in tame rats to that in aggressive rats (hereinafter: log2-value) within an orthologous DEG pair being tested (STEP 3). Finally, using both linear and rank correlation analysis, we found significant positive correlations between fold changes of the multiple homologous DEGs (PC1) and significant negative correlations between fold changes of the only single homologous DEGs (PC2); this is STEP 4 in Figure 1.

### 2.6. Statistical Analysis

As depicted in Figure 1, during our statistical analysis, we compiled standard statistical 2 × 2 tables, which served as input data for the standard software Statistica (Statsoft^TM^, Tulsa, OK, USA) in its pipeline “Statistics” → “Nonparametric” → “Table 2 × 2.” This brought us to Fisher’s exact test, Pearson’s χ^2^ test, and binomial distribution analysis to test the significance of the results (Figure 1). Likewise, by means of the same toolbox with proper options, we carried out the Mann–Whitney *U* test, Fisher’s Z-test (see Section 2.4), and principal component analysis (Figure 1).

## 3. Results

### 3.1. RNA-Seq and Mapping to the Reference Rat Genome

Using an Illumina NextSeq 550 system, we sequenced the hypothalamus transcriptome of three tame adult male rats and that of three aggressive ones. The rats had no family relations and represented two outbred lines (see Section 2). From 219,086,104 raw reads (75 nt, deposited in the NCBI, PRJNA668014) 184,991,379 reads (84%) were mapped to reference genome Rn6 (Table 3). This allowed us to identify 14,039 genes expressed within the hypothalamus of adult male rats under the experimental conditions used (Table 3). According to Fisher’s Z-test, 1025 of these genes (7%) were statistically significantly differentially expressed between tame and aggressive rats in their hypothalamus at the commonly accepted confidence threshold *p* < 0.05, as presented in Table 3. To minimize false positive error rates, we applied the Benjamini correction for multiple comparisons, which finally resulted in 46 DEGs in the hypothalamus of the tame versus aggressive rats under study (Table 3); the DEGs are listed in Table 4. In particular, rat gene *Ascl3* encoding achaete-scute family bHLH transcription factor 3 turned out to be the best DEG within the hypothalamus of the tame versus aggressive rats owing to its smallest P_ADJ_ value, 10^−8^. This DEG has a log2 value of 3.99, as shown in Table 4, row #1.

### 3.2. qPCR Selective Verification of the DEGs Identified in this Work in the Hypothalamus of Tame versus Aggressive Rats

For this purpose, we selected three out of the 46 DEGs listed in Table 4, namely, *Ascl3* (i.e., the above-mentioned best DEG), *Apobec1*, and *Defb17*, as shown in Table 5.

Additionally, separately, we prepared eight other tame adult male rats and eight other aggressive ones (all unrelated), who scored either “−3” or “3” (Table 5) on a scale from −4 (the most aggressive rat) to 4 (the tamest rat) in the standard glove test [20]. It was performed 1 month before the extraction of hypothalamus samples to minimize the effects of the glove test on the results of this study (see Section 2). At the bottom of Table 5, we present our qPCR data on the three DEGs examined in the hypothalamus of the aggressive versus tame rats as mean ± standard error of mean (M_0_ ± SEM) of their relative expression with respect to four reference genes *B2m*, *Hprt1*, *Ppia*, and *Rpl30* on the basis of three technical replicates. These qPCR values (expression levels) varied from 0.01 to 9.22. In three out of eight aggressive rats (#3, #4, and #5), *Defb17* expression levels in the hypothalamus were below the threshold of sensitivity under the experimental conditions where we reliably measured the expression of all the tested genes in all studied rats (except for these three cases, as indicated by “ND, not detected” in Table 5).

**Figure 2:**
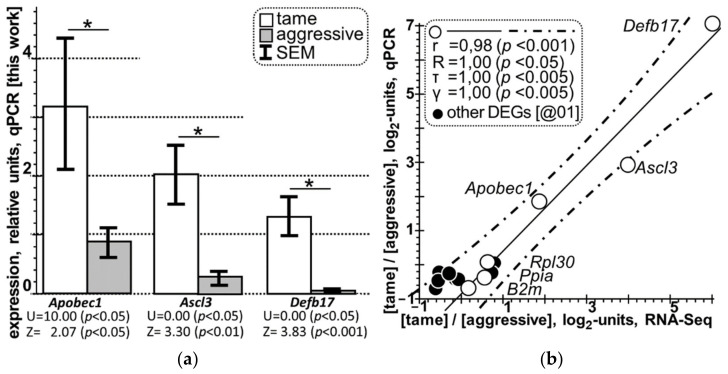
Selective qPCR-based verification of the DEGs identified in this work in the hypothalamus of tame versus aggressive rats. (**a**) In tame rats (white bars) versus aggressive rats (grey bars), all the three DEGs examined (i.e., *Ascl3*, *Apobec1,* and *Defb17*) are statistically significantly overexpressed in the hypothalamus (here, bar height: mean; error bars: standard error of mean [SEM]; and asterisks (i.e., the characters "*") denote statistical significance at *p* < 0.05 according to both the Mann–Whitney *U* test and Fisher’s Z-test). (**b**) Statistically significant correlations between the log2 value of the three selected DEGs and three reference genes [i.e., *B2m*, *Ppia*, and *Rpl30*] in the hypothalamus of tame versus aggressive rats (open circles), as measured experimentally by RNA-Seq (*X*-axis) and qPCR (*Y*-axis). Solid and dash-and-dot lines denote linear regression and boundaries of its 95% confidence interval calculated by means of software package Statistica (Statsoft^TM^, Tulsa, OK, USA). Statistics: r, R, τ, γ, and *p* are coefficients of Pearson’s linear correlation, Spearman’s rank correlation, Kendall’s rank correlation, Goodman–Kruskal generalized correlation, and their *p* values (statistical significance), respectively. Filled circles depict the statistically significant DEGs in the hypothalamus of tame vs. aggressive rats according to the independent qPCR-based identification published elsewhere [34]. These qPCR [34] and RNA-Seq [this work] data are given in Table S1, where their Pearson’s linear correlation is statistically significant, r = 0.71 at *p* < 0.05 (hereinafter, see Supplementary Materials).

Moreover, in the rightmost column of Table 5, we present the results of averaging the expression levels of each of the three verified DEGs in the hypothalamus of the aggressive and tame rats examined. Figure 2a shows graphical representation of these qPCR results. 

In this figure, readers can see statistically significant overexpression of all the three examined DEGs in the hypothalamus of the tame male adult rats (white bars) compared with aggressive ones (grey bars) according to the qPCR data obtained in this work, in agreement with the expression levels detected in the transcriptome analysis (Table 4), as depicted by asterisks, each of which means *p* < 0.05 in both the Mann–Whitney *U* test and Fisher’s Z-test, which are independent from one another. 

Figure 2b presents statistically significant correlations between relative expression levels in the hypothalamus of tame versus aggressive rats, as measured experimentally by two methods [RNA-Seq (*X*-axis) and qPCR (*Y*-axis)] independent from each other, with the results expressed in log2 values (see “Materials and methods”) for the six genes, namely: three selected DEGs (i.e., *Ascl3*, *Apobec1*, and *Defb17*) and three reference genes (i.e., *B2m*, *Ppia,* and *Rpl30*) as depicted by open circles. In this figure, solid and dash-and-dot lines represent linear regression and boundaries of its 95% confidence interval, as calculated by means of software package Statistica (Statsoft^TM^, Tulsa, OK, USA). As one can see, the coefficients of Pearson’s linear correlation (r = 0.89, *p* < 0.001), Spearman’s rank correlation (R = 1.00, *p* < 0.05), Kendall’s rank correlation (τ = 1.00, *p* < 0.005), and Goodman–Kruskal generalized correlation (γ = 1.00, *p* < 0.005) are statistically significant, while being independent from one another.

Moreover, the filled circles depict seven statistically significant DEGs in the hypothalamic samples of eight other tame rats versus eight other aggressive rats according to a qPCR-based identification protocol published in our previous work [34]. These RNA-Seq [this work] and qPCR [34] data are given in Table S1, where their Pearson’s linear correlation is statistically significant too, namely: r = 0.71 at *p* < 0.05 (hereinafter, see Supplementary Materials). Moreover, as readers can see here, all the seven independently identified DEGs do not go beyond the boundaries of the 95% confidence interval of the linear regression under consideration (i.e., they are between the two dash-and-dot lines).

Summing Figure 2 up, both the nonparametric Mann–Whitney *U* test and parametric Fisher’s Z-test as well as Pearson’s linear correlation, Goodman–Kruskal generalized correlation, and Spearman’s and Kendall’s rank correlations taken together independently indicate that in this study, the qPCR data on the hypothalamus of eight tame adult male rats compared with eight aggressive ones statistically significantly confirm the DEGs found in the hypothalamus of three other tame adult male rats versus three other aggressive ones (all unrelated animals). This confirmation means statistical robustness of the DEGs identified in this work.

### 3.3. Verification of the DEGs Found Here in the Hypothalamus of Tame versus Aggressive Rats with Respect to Their Known Homologous DEGs in Domestic and Wild Animals (All Data That We Could Find)

In this study, we for the first time verified the DEGs identified by us in the hypothalamus of tame versus aggressive rats with respect to their known homologous DEGs in domestic animals compared with their wild congeners as reported by others (Figure 1 and description in Section 2). With this in mind, to the 46 above-mentioned hypothalamic DEGs of tame versus aggressive adult male rats (Table 4), we first of all added 14 independent publicly available RNA-Seq datasets on domestic versus wild animals [23,24,25,26,27,28,29], entitled “TEST DATA” in Figure 1 and characterized in Table 2. There, to minimize false positive error rates, we took into account only the statistically significant DEGs (according to the Benjamini correction for multiple comparisons) published in relevant articles cited in the rightmost column of Table 2. This procedure eventually resulted in 2347 DEGs in seven tissues of seven pairs of domestic versus wild cognate animals (Table 2: bottom row).

As depicted by a Venn diagram in Figure 1 (STEP 1), next, we managed to compile 54 pairs of homologous DEGs (listed in Table 6), where each pair contains one DEG taken from Table 4 (i.e., columns i to iv of Table 6) as well as its homologous DEG chosen from the 2347 DEGs characterized in Table 2 as detailed in columns v to xi of Table 6. 

**Figure 3:**
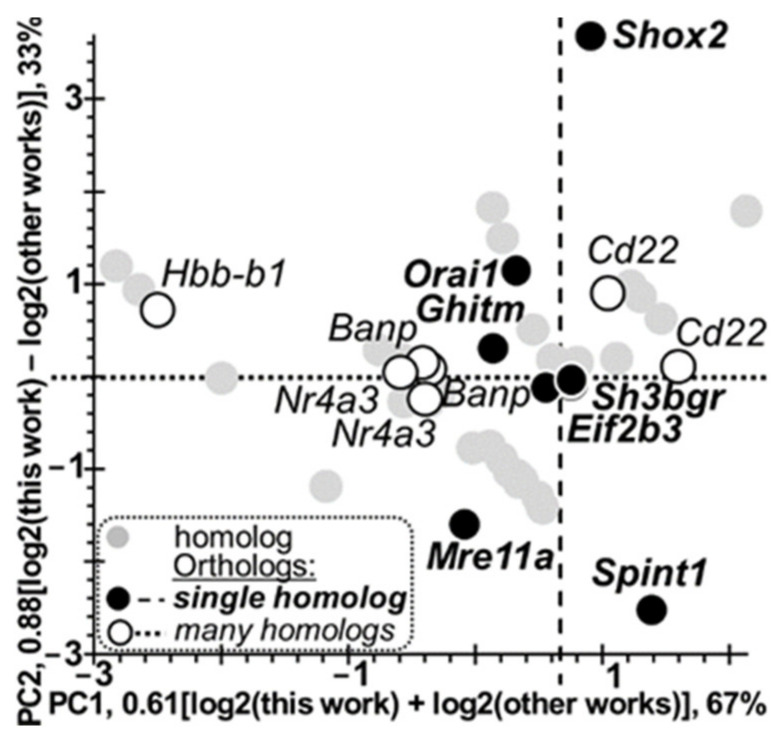
The results of principal component analysis of the hypothalamic DEGs of tame versus aggressive rats found in this work in comparison with their known homologous DEGs in domestic animals versus their wild congeners as reported by others. Legend: see the footnote of Table 4 and legend of Figure 1; open circles: all seven single orthologs *Eif2b3* (Table 6: row #6), *Ghitm* (#9), *Mre11a* (#28), *Orai1* (#34), *Sh3bgr* (#51), *Shox2* (#52), and *Spint1* (#53) grouped along a dashed line; filled circles: all seven orthologs supported by other homologs *Banp* (#1 and #2, which support one another in homology), *Cd22* (#3 and #4 supporting each other in homology), *Nr4a3* (#29 and #30 supported one another in homology as well as by their paralogs in rows ## 31–33), and *Hbb-b1* (#10 supported by its paralogs in rows ## 11–14) grouped along a dotted line; grey circles: all the remaining homologs; log2 (this work) and log2 (other works) correspond to columns iii and ix of Table 6; PC1 and PC2: first (major) and second (minor) principal components calculated by means of software package Statistica (Statsoft^TM^, Tulsa, OK, USA); they are parallel to the dotted and dashed lines, respectively.

Following our previous article [36] on a factor analysis of qPCR-identified DEGs within in the hypothalamus of tame versus aggressive rats [34], in this work, we processed Table 6 by principal component analysis (Figure 1: STEP 2), the results of which are presented in Figure 3. In this figure, one can see that the first (main) principal component PC1 (*X*-axis) is proportional to arithmetic means of the log2 values measured by independent experiments on domestic animals compared with their wild congeners. PC1 explains two-thirds (67%) of differential-gene-expression variance under study. This is parallel to the dotted line (Figure 3) along which all orthologs (*Banp*, *Cd22*, *Nr4a3*, and *Hbb-b1*) with multiple homologs are located. Indeed, two *Banp*-related orthologous gene pairs (#1 and #2) supported one another in homology, as did two *Cd22*-related gene pairs (#3 and #4). Additionally, two *Nr4a3*-related gene pairs (#29 and #30) were supported by their paralogs (rows ## 31–33), as was the *Hbb-b1*-related pair (#10); it is supported by its paralogs in rows ## 11–14. Additionally, the second (minor) principal component, PC2 (Figure 3: *Y*-axis), is proportional to the difference between the estimates determined by the measurement in this work and corresponding estimates obtained independently by others. PC2 explains one-third (33%) of differential-gene-expression variance under study. It is parallel to the dashed line, where all single orthologs (*Eif2b3*, *Ghitm*, *Mre11a*, *Orai1*, *Sh3bgr*, *Shox2*, and *Spint1*) are located (Table 6: rows ## 6, 9, 28, 34, 51, and 52, respectively).

After that, keeping the two pairs of parallel lines in Figure 3 in mind (i.e., PC1 and the dotted line fitting orthologous DEGs with multiple homologs as well as PC2 and the dashed line fitting single orthologous DEGs), we performed a statistical analysis of the binary correlation between either multiple-homolog orthologous DEGs or single orthologous DEGs, namely, we tested whether there are the same or opposite signs of the log2 value within an orthologous DEG pair being tested (Figure 1: STEP 3).

To this end, we first formatted standard Fisher’s 2 × 2 table for binary correlation tests and then performed Fisher’s exact test, Pearson’s χ^2^ test, and binomial distribution analysis using the standard software, Statistica (Statsoft^TM^, Tulsa, OK, USA), as shown in Table 7. Within columns iii and iv of this table, readers can see that all the seven orthologous DEG pairs supported by multiple homologs have the same sign, in contrast to most of single orthologous DEG pairs (five of seven), which have opposite signs. This difference between single orthologous DEGs and those supported by multiple homologs is statistically significant according to Pearson’s χ^2^ test and Fisher’s exact test, as shown in columns vii and v of Table 7, respectively. Moreover, according to binomial distribution analysis (column v of Table 7), the orthologous DEG pairs supported by multiple homologs statistically significantly keep the sign of the log2 value in independent experiments on domestic versus wild animals (*p* < 10^−3^), whereas single orthologous DEGs are statistically insignificant in this regard (*p* > 0.2). Thus, the set of the DEGs identified by other authors in their experiments on domestic versus wild animals (Table 2) contains at least two biologically different subsets, namely: (a) PC1-linked orthologous DEGs supported by multiple homologs and (b) PC2-linked single orthologous DEGs.

**Figure 4:**
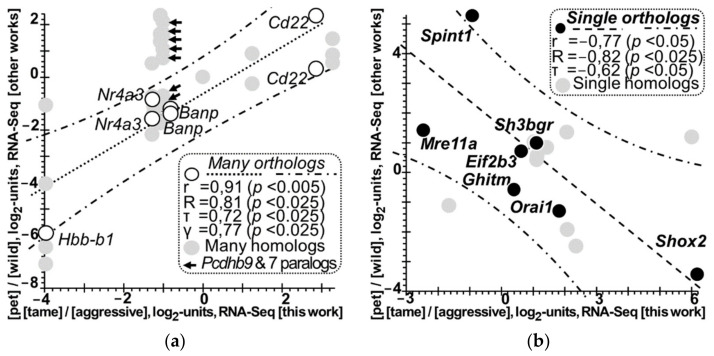
Statistically significant correlations between the log2 values of DEGs in the hypothalamus of tame vs. aggressive rats as measured in this work (*X*-axis; column iii of Table 6) and the log2 values of their known orthologous DEGs in domestic vs. wild animals as measured by others (*Y*-axis; column ix of Table 6). Legend: see legends of Figure 2 and Figure 3; (**a**) PC1-linked orthologous DEGs supported by multiple homologs; arrows (→) point to *Pcdhb9* compared with its seven paralogs (rows ## 36–42 of Table 6); (**b**) PC2-linked single orthologous DEGs.

Therefore, we finally performed a correlation analysis on the DEGs found in this work (in the hypothalamus of tame versus aggressive rats) with respect to both the PC1- and PC2-linked orthologous DEGs mentioned above (Figure 1: STEP 4) independently from one another, as readers can see in Figure 4a,b, respectively. As shown in Figure 4a, according to four statistical criteria—i.e., Pearson’s linear correlation (r), the Goodman–Kruskal generalized correlation (γ), and Spearman’s (R) and Kendall’s (τ) rank correlations—there are statistically significant positive correlations between the log2 values of DEGs in the hypothalamus of tame versus aggressive rats (*X*-axis; column iii of Table 6) [this work] and the log2 values of their orthologous DEGs (in domestic versus wild animals as measured by others: *Y*-axis; column ix of Table 6) supported by multiple homologs (i.e., PC1-linked).

Moreover, gray circles in this figure show similar pairs formed by DEGs identified here and their homologous DEGs identified by others and supported by multiple homologs (i.e., PC1-linked too). These circles fit reasonably well into the 95% confidence interval (between two dash-and-dot lines) of the linear correlation presented (a dotted line). 

In Figure 4a, arrows point to the rat gene *Pcdhb9* compared with its seven paralogs (rows ## 36–42 of Table 6), where two of these seven pairs fit within the 95% confidence interval (rows ## 36 and 38 in Table 6), while five remaining pairs fall outside this interval. Because the paralogous genes are homologs that arose via a duplication of their common ancestral gene and next via divergence in their biological functions, the observed partial deviation of these paralogous pairs from the common pattern of the genes-orthologs keeping functions from species to species looks expected rather than unexpected. 

All these robust correlations taken together allow us for the first time to identify common orthologous DEGs of various domestic animals (versus their wild congeners) that from species to species and from tissue to tissue, statistically significantly keep the sign of the log2 value in line with principal component 1 of this domestication-related differential gene expression. PC1 explains two-thirds (67%) of this expression pattern variance (Figure 3 and Figure 4a). This finding may reflect the most common pattern of animal domestication.

Finally, Figure 4b presents significant negative correlations between the log2 values measured in this work (*X*-axis; column iii of Table 6) and those measured in experiments by others in domestic versus wild animals (*Y*-axis; column ix of Table 6) in the case of the PC2-linked single orthologous DEGs; these correlations are statistically significant according to Pearson’s linear correlation (r) and Spearman’s (R) and Kendall’s (τ) rank correlation analyses. In addition, gray circles depict similar single pairs of DEGs identified here with their homologous DEGs identified elsewhere (i.e., PC2-linked too) that fit reasonably well between two dash-and-dot lines, where the 95% confidence interval covers the linear correlation examined (a dotted line). Altogether, this robust evidence for the first time shows that some unique orthologous DEGs of various domestic versus wild animals can statistically significantly reverse the log2 value sign in agreement with the principal component (PC2) explaining one-third (33%) of this domestication-related differential-gene-expression variance (Figure 3 and Figure 4b). Indeed, this is exactly what we have already seen in both qPCR and the maximum-variation (maxVar) factor analysis [35], namely: two factors of the differential gene expression (in tame versus aggressive rats) that explain 67% of its variance: behavioral selection and laboratory lifestyle (i.e., domestication), while the remaining 33% of the variance can be explained by intraspecific variation [35]. Therefore, here the second principal component (PC2) explaining 33% of the differential gene expression variance between domestic and wild animals (Figure 3 and Figure 4b) reflects both interspecific variation and intraspecific variation and is close to the gold standard of variation in nature. 

Thus, here we for the first time simultaneously quantified two independent phenomena—domestication [1] and cladogenesis [6]—at the same scale (genome-wide analysis). These phenomena respectively explained two-thirds and one-third of differential-gene-expression variance between domestic and wild animals as responses of their genomes to effects of artificial and natural environments. Nevertheless, it seems that the elucidation of the influence of both interspecific and intraspecific variation on the process of domestication of animals by humans requires further research, which is outside the scope of this work.

## 4. Discussion

**Figure 5:**
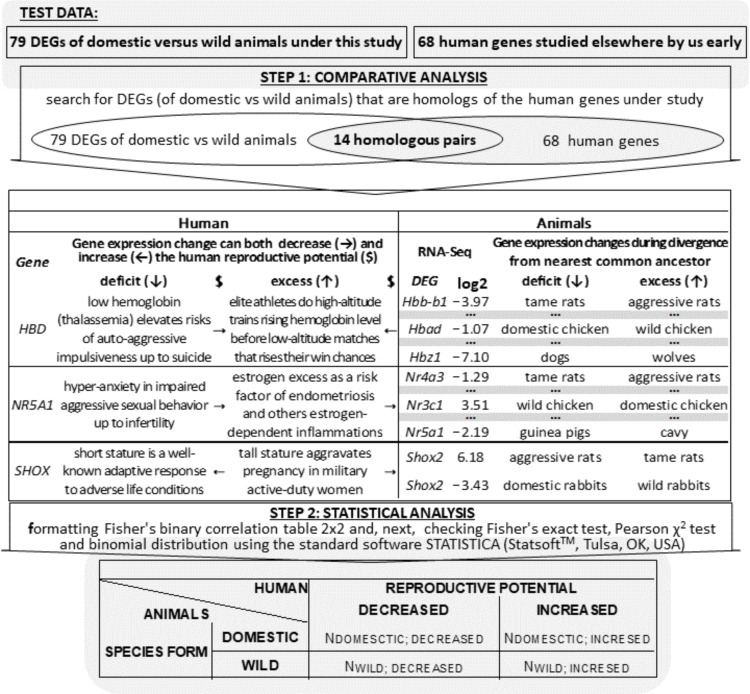
An algorithmic flowchart depicting biomedical application of our results to the search for human candidate genes contributing to self-domestication syndrome. Legend: see the footnotes of Table 4 and Table 6 as well as legends of Figure 1, Figure 2 and Figure 3.

Let us discuss biomedical applications of the results, keeping in mind that the mainstream in genome-wide studies on domestic versus wild animals is the search for species-specific DEGs [23,24,25,26,27,28,29]. There is an exception where investigators introduced the new biomedical concept of self-domestication syndrome [30], although the idea of human self-domestication is still debatable [31]. An algorithmic flowchart illustrating the biomedical application of our results to the search for human candidate genes contributing to self-domestication syndrome is presented in Figure 5. As readers can see in this figure, the test data under study included the 54 homologous DEG pairs of domestic versus wild animals (Table 6), where the total number of animal genes is 79.

Additionally, we took into account all the 68 human genes whose effects on human reproductive potential (as the most common index of how many chances humans have to survive, have children, and help them become the next generation under the best conditions [53,54]) have previously been estimated elsewhere [55,56] by means of SNPs within human gene promoters. In the present study, we updated these estimates in line with the current state of PubMed [52], as shown in Table S2.

First of all, using the above-mentioned test data, we compiled all possible (14) pairs of the human genes together with their homologous genes in animals, as depicted by a Venn diagram in Figure 5 (STEP 1). Table 8 presents these pairs of homologous human versus animal genes as follows: the left half of this table (i.e., columns i to v) is a copy of the data on a human gene in question from Table S2, whereas the right half (i.e., columns vi, vii, and x) is a copy of the RNA-Seq data on the corresponding homologous DEG in domestic versus wild animals (Table 6).

Furthermore, columns viii and ix of Table 8 translate a log2 value of a gene in an animal into either low or excessive expression of this gene during divergence of both domestic and wild forms of this animal from their most recent common ancestor. Recently [57,58], an RNA-Seq data analysis was based on this oldest phylogeny concept [59] widely used elsewhere [60,61,62,63]. Let us look at these orthologous genes one by one and discuss effects of their expression changes on reproductive potential in humans as well as during divergence of domestic and wild animals from their most recent com-mon ancestor [24,25,28,29,64,65,66,67,68,69,70,71,72,73,74,75,76,77,78,79,80,81,82,83,84,85,86] (Table 8).

Human gene *HBD* produces hemoglobin subunit δ, a deficit of which (thalassemia) is clinically proven as a risk factor for auto-aggressive impulsiveness up to suicide [64], female subfertility [65], under-threshold IQ, and severe anxiety in children [66], as readers can see in Table 8. Actually, both suicide and IQ look like human-specific traits, which are biomedically studied mostly in human behavioral models based on animals [67,68] and are not common in the wild [56]. Curiously, anxiety is the most important trait for mutual trust within a human–pet pair, as independently discovered for dogs [69], sheep [70], and guinea pigs [71]. Moreover, according to a sports medicine report [72], combat success of healthy young boxers, kick boxers, and karate fighters increases with an increase in their anxiety in the arena (this anxiety prevents injuries until the end of a fight or sparring).

**Table 8:** A comparison of the effects of changes in the expression of orthologous genes on human reproductive potential through aggressiveness changes and on various traits during the divergence of domestic animals and their wild congeners from the corresponding most recent common ancestor.

Humans					Animals				
*Gene*	Effect of Gene Expression Changes on Human Reproductive Potential, Namely ($): Decreased (→) or Increased (←)				RNA-Seq		Effect of Gene Expression Changes during DIVERGENCE from the most Recent Common Ancestor		[Ref]
	Deficit (↓)	$	Excess (↑)	$	*DEG*	log2	Deficit (↓)	Excess (↑)	
i	ii	iii	iv	v	vi	vii	viii	ix	x
*HBD*	hemoglobin deficit (thalassemia) elevates risks of auto-aggressive impulsiveness up to suicide [64], female subfertility [65], causes under-threshold IQ and severe anxiety in children [66]	**→**	in cohort studies: elite athletes do high-altitude training rising hemoglobin level before low-altitude matches thereby increasing their chances of winning [76]	**←**	*Hbb*-*b1*	−3.97	tame rat	aggressive rat	[75]
					*Hbbl*	−5.92	dogs	wolves	[24]
					*Hba1*	−4.06	dogs	wolves	[24]
					*Hbad*	−1.07	domestic chicken	wild chicken	[29]
					*Hbm*	−6.46	dogs	wolves	[24]
					*Hbz1*	−7.10	dogs	wolves	[24]
*NR5A1*	within human disease models based on *Nr5a1*-null male mice: hyper-anxiety during impaired aggressive sexual behavior up to male infertility in line with male patients carrying NR5A1 defects [77] as well as NR5A1 deficit can cause hypoestrogenism [78] leading to 1% of cases of female infertility [79]	**→**	in retrospective meta-analysis of PubMed content: NR5A1 excess contributes to excessive estrogen biosynthesis raising risks of estrogen-dependent inflammatory disorders in women [80] and vice versa for men [81]	**→**	*Nr4a3*	−1,29	tame rat	aggressive rat	[82]
					*Nr4a3*	−0.85	domestic chicken	wild chicken	[29]
					*Nr4a3*	−1.58	domestic rabbits	wild rabbits	[28]
					*Nr2c1*	−0.74	guinea pigs	cavy	[25]
					*Nr3c1*	0.51	wild chicken	domestic chicken	[29]
					*Nr5a1*	−2.19	guinea pigs	cavy	[25]
*SHOX*	in cohort studies: low SHOX expression causes short stature [83] as adaptive epigenetic response to adverse living conditions, when each calorie saved due to short stature helps to enhance stress resistance [84]	**←**	in cohort studies: girls carrying one extra *SHOX* copy have tall stature [85] elevating risks of pregnancy complications in military active-duty women [86]	**→**	*Shox2*	6.18	aggressive rat	tame rat	[87]
					*Shox2*	−3.43	domestic rabbits	wild rabbits	[28]

Finally, via Stroop-like interference effect approximation [73], Nobel laureate Daniel Kahneman [74] highlighted anxiety among pivotal factors for human economic decision making during exposure to both psychological and social stressors. As shown in Table 8, a human hemoglobin deficit is consistent with that in tame rats [75], dogs [24], and domestic chickens [29] during their divergence from the most recent common ancestors along with their wild congeners (column viii) according to their negative log2 values (column vii). 

As for human hemoglobin overexpression, according to a cohort-based study [76], elite athletes do high-altitude training raising the hemoglobin level before low-altitude matches thereby increasing their chances of winning. Thus, humans’ chances for success increase with a small subcritical increase in their hemoglobin level, consistently with a hemoglobin excess in aggressive rats [75], wolves [24], and wild chickens [29] during their microevolution (Table 8: column ix) according to their positive log2 values (Table 8: column vii). Altogether, readers can see in row #1 of Table 8 that animal genes that are homologs of the human *HBD* gene, from species to species are underexpressed in domestic animals and overexpressed in their wild congeners with respect to their most recent common ancestors. 

This finding fits the first principal component (PC1) explaining two-thirds (67%) of the differential-gene-expression variance during the domestication-related microevolution (Figure 3 and Figure 4a). With this in mind, we suggest *HBD* as a candidate gene contributing to self-domestication syndrome, namely: low HBD expression might be regarded as what humans pay with health (e.g., higher risks of suicide [64], female subfertility [65], low IQ, and anxiety in children [66]) for the benefits received during evolution (e.g., the ability to build trust [69,70,71], prevention of injuries [72], and making adequate decisions under stress [74]).

Human gene *NR5A1* encodes steroidogenic factor 1, both a deficit and excess of which reduce human reproductive potential on the one hand via hyper-anxiety during impaired aggressive sexual behavior up to male infertility [77] as well as hypoestrogenism [78] leading to 1% of cases of female infertility [79], and on the other hand, hyperestrogenism elevates risks of endometriosis and others estrogen-dependent inflammatory disorders in women [80,81]. That is why both domestic and wild forms of rats [82], chickens [29], rabbits [28], and guinea pigs [25] could have paid with their health (i.e., hormonal dysregulation) for the benefits that each of them got during microevolution (Table 8). Thus, we have no idea whether *NR5A1* has something to do with self-domestication syndrome.

Human gene *SHOX* (short stature homeobox) underexpression is an adaptive epigenetic response to adverse living conditions in humans according to a cohort-based study [83] because each calorie saved due to short stature helps to enhance stress resistance [84]. This *SHOX* insufficiency in humans corresponds to a *Shox2* deficit [87] in aggressive rats under study. Nonetheless, in another cohort-based study [85], researchers analyzed girls carrying one extra *SHOX* copy manifesting in tall stature, which is a risk factor for pregnancy complications in military active-duty women [86]. As readers can see in Table 4, Table 6, and Table 8, within the framework of our study, the human *SHOX* overexpression matches *Shox2* overexpression [87] in tame rats. Let us recall Figure 3 and Figure 4b, where the animal *Shox2* gene was seen within only a single orthologous pair of genes carrying opposite signs of log2 values compared with each other, in accordance with the second principal component (PC2) explaining one-third (33%) of the differential-gene-expression variance during domestication. Therefore, in contrast to tame rats, domestic rabbits seem to do well with short stature [83] because they could be anthropogenically artificially selected for the purpose of increasing their fertility for meat production, despite the tall-stature–related complications of pregnancy [85,86], as shown in the last row of Table 8. With this in mind, we would link the second principal component illustrated here by the animal *Shox2* gene (Figure 3 and Figure 4b) to both interspecific and intraspecific variation.

Altogether, here we propose *SHOX* as a candidate human gene contributing to self-domestication syndrome as follows: *SHOX* excess as an epigenetic response to better life [84] resulting in tall stature [83] might be regarded as what humans pay with health (e.g., the tall-stature–related complications of pregnancy [85,86]) for the benefits received during evolution (e.g., the anthropogenic environment instead of the natural one).

Table 9 is standard Fisher’s 2 × 2 table that summarizes the findings of the comparative analysis of the above-mentioned similar genes of humans and animals (columns iii and iv) and presents the results of statistical analysis of these data, as shown in Figure 5 (STEP 2). As evident here, 13 and one of these domestic-animal DEGs correspond to human genes-markers of a decrease and increase in human reproductive potential, while the same is true for seven and seven DEGs in the wild animals.

Therefore, one can see that the DEGs in domestic animals statistically significantly match their human orthologous genes aggravating human diseases according to Pearson’s χ^2^ test (*p* < 0.05), Fisher’s exact test (*p* < 0.05), and binomial distribution analysis (*p* < 0.001). Finally, the last row of this table illustrates that DEGs of wild animals correspond to a set of human orthologous genes where some genes weaken while others improve human reproductive potential (*p* > 0.5, binomial distribution), overall corresponding to a norm (i.e., the wild type). Thus, the artificial environment of domestic animals alters the activity of their genes in the same direction as that seen in the corresponding human genes during some human diseases, whereas the natural environment maintains a normal gene expression pattern in wild animals (corresponding to health in humans).

## 5. Conclusions

First, in this study, we sequenced the hypothalamus transcriptome of tame and aggressive adult male rats and deposited these primary experimental data in the NCBI SRA database [40] (ID = PRJNA668014), where they are freely available for those who would like to use them in the future. 

Second, in these data, we identified 46 DEGs (in the hypothalamus of the tame versus aggressive rats under study) that were statistically significant (P_ADJ_ < 0.05, according to correction for multiple comparisons). We selectively verified the reproducibility of these DEGs in another qPCR experiment on an independent set of biological samples. 

Third, using principal component analysis, we for the first time compared the 46 hypothalamic DEGs of tame versus aggressive rats found here with 2347 DEGs of domestic versus wild animals found by others. This analysis yielded two principal components, PC1 and PC2, respectively explaining 67% and 33% of the differential-gene-expression variance between all the domestic and wild animals under study. In this way, we showed that PC1 corresponds mostly the orthologous DEGs supported by multiple homologs, which often kept the sign of their log2 values from species to species and from tissue to tissue as the common pattern of animal domestication. On the contrary, PC2 corresponds to the single orthologous DEGs without homologous supporting genes; these DEGs mainly reversed the sign of their log2 values from one species to another and from tissue to tissue; thus, PC2 may reflect both intraspecific and interspecific variation of gene expression alterations during domestication. This allows us to conclude that domestication explains two-thirds of differential-gene-expression variance between domestic and wild animals (i.e., PC1), whereas the remaining one-third reflects intraspecific and interspecific variation (i.e., PC2).

## Figures and Tables

**Table 1:** For quantitative polymerase chain reaction (qPCR), primers were selected using Web service PrimerBLAST [47].

No.	Rat Gene	NCBI Gene ID	Direct, 5′→3′	Reverse, 5′→3′
i	ii	iii	iv	v
**DEGs Identified** **in Hypothalamus of Tame versus Aggressive Rats [This Work]**				
1	*Apobec1*	*25383*	CGCCGCAACATAAGCTCCCGA	TGCTGTGCCTTCCTCCCCAGTTG
2	*Ascl3*	*246301*	CCTCTGCTGCCCTTTTCCAG	ACTTGACTCGCTGCCTCTCT
3	*Defb17*	*641658*	TGGTAGCTTGGACTTGAGGAAAGAA	TGCAGCAGTGTGTTCCAGGTC
**Reference Genes**				
4	*B2m*	*24223*	GTGTCTCAGTTCCACCCACC	TTACATGTCTCGGTCCCAGG
5	*Hprt1*	*24465*	TCCCAGCGTCGTGATTAGTGA	CCTTCATGACATCTCGAGCAAG
6	*Ppia*	*25518*	TTCCAGGATTCATGTGCCAG	CTTGCCATCCAGCCACTC
7	*Rpl30*	*64640*	CATCTTGGCGTCTGATCTTG	TCAGAGTCTGTTTGTACCCC

Notes. The rat differentially expressed genes (DEGs) subjected to this qPCR verification: *Apobec1*, apolipoprotein B mRNA editing enzyme catalytic subunit 1; *Ascl3*, achaete-scute family bHLH transcription factor 3; *Defb17*, defensin β 17; *B2m*, β-2-microglobulin [48]; *Hprt1*, hypoxanthine phosphoribosyltransferase 1 [49]; *Ppia*, peptidylprolyl isomerase A [50]; and *Rpl30*, ribosomal protein L30 [51].

**Table 2:** The investigated genome-wide transcriptomes of domestic versus wild animals, all the data that we could find in PubMed [52].

#	Wild Animals	Domestic Animals	Tissue	N_DEG_	[Ref]
1	aggressive foxes (*Vulpes vulpes*)	tame foxes (*V. vulpes*)	pituitary	327	[23]
2	wolves (*Canis familiaris*)	dogs (*C. lupus*)	blood	450	[24]
3	wolves (*C. lupus*)	dogs (*C. familiaris*)	frontal cortex	13	[25]
4	Boars (*Sus scrofa*)	pigs (*S. scrofa*)	frontal cortex	30	[25]
5	cavy (*Cavia aperea*)	guinea pigs (*C. porcellus*)	frontal cortex	883	[25]
6	wild rabbits (*Oryctolagus cuniculus*)	domestic rabbits (*O. cuniculus domesticus*)	frontal cortex	17	[25]
7	aggressive rats (*R. norvegicus*)	tame rats (*Rattus norvegicus*)	frontal cortex	20	[25]
8	boars (*S. scrofa*)	pigs (*S. scrofa*)	frontal cortex	34	[26]
9	boars (*S. scrofa*)	pigs (*S. scrofa*)	pituitary	22	[27]
10	wild rabbits (*O. cuniculus*)	domestic rabbits (*O. cuniculus domesticus*)	parietal-temporal cortex	216	[28]
11	wild rabbits (*O. cuniculus*)	domestic rabbits (*O. cuniculus domesticus*)	amygdala	118	[28]
12	wild rabbits (*O. cuniculus*)	domestic rabbits (*O. cuniculus domesticus*)	hypothalamus	43	[28]
13	wild rabbits (*O. cuniculus*)	domestic rabbits (*O. cuniculus domesticus*)	hippocampus	100	[28]
14	wild chicken (*G. gallus*)	domestic chicken (*Gallus gallus*)	pituitary	474	[29]
Σ	7 wild animal species:	7 domestic animal species:	7 tissues	2347	7 Refs

Note: N_DEG_, number of DEGs; Ref, reference.

**Table 3:** A summary of transcriptome sequencing in the hypothalamus of three tame rats and three aggressive ones (all unrelated).

Group	Tame vs. Aggressive Rats
Total sequence reads (NCBI SRA, ID = PRJNA668014)	219,086,104
Reads mapped to reference rat genome RGSC Rnor_6.0, UCSC Rn6, July 2014 (%)	184,991,379 (84.44%)
Expressed genes identified	14,039
Candidate DEGs identified (*p* value < 0.05; Fisher’s Z-test)	1025
Statistically significant DEGs (P_ADJ_ < 0.05, Benjamini correction)	46

**Table 4:** The statistically significant DEGs in the hypothalamus of tame versus aggressive rats bred artificially during 90 generations from the same common ancestral subpopulation.

Rat Gene			Differential Expression		
No.	Symbol	Name	log2	*p*	*P_ADJ_*
*1*	*Ascl3*	achaete-scute family bHLH transcription factor 3	3.99	10^−12^	10^−8^
*2*	*Morn1*	MORN repeat containing 1	1.24	10^−10^	10^−6^
*3*	*Krt2*	keratin 2	−1.65	10^−8^	10^−4^
*4*	*Banp*	Btg3 associated nuclear protein	−0.82	10^−6^	10^−3^
*5*	*Mre11*	MRE11 homolog, double strand break repair nuclease	−2.47	10^−6^	10^−3^
*6*	*Rbm3*	RNA binding motif protein 3	1.04	10^−6^	10^−3^
*7*	*Fcgr3a*	Fc fragment of IgG receptor IIIa	2.06	10^−6^	10^−2^
*8*	*Plac8*	placenta associated 8 (synonym: onzin)	2.83	10^−5^	10^−2^
*9*	*Cd22*	CD22 molecule	2.85	10^−5^	10^−2^
*10*	*Apobec1*	apolipoprotein B mRNA editing enzyme catalytic subunit 1	1.87	10^−5^	10^−2^
*11*	*Magee2*	MAGE family member E2	−0.95	10^−5^	10^−2^
*12*	*Hbb-b1*	hemoglobin, β adult major chain	−3.97	10^−5^	10^−2^
*13*	*Tpm2*	tropomyosin 2	1.44	10^−5^	10^−2^
*14*	*Apobr*	apolipoprotein B receptor	1.56	10^−5^	10^−2^
*15*	*Cenps*	centromere protein S	1.63	10^−4^	0.05
*16*	*Gale*	UDP-galactose-4-epimerase	1.15	10^−4^	0.05
*17*	*Pcdhb9*	protocadherin β9	−1.01	10^−4^	0.05
*18*	*P2rx4*	purinergic receptor P2X 4	1.14	10^−4^	0.05
*19*	*Rn45s*	45S pre-ribosomal RNA	−1.62	10^−4^	0.05
*20*	*Nr4a3*	nuclear receptor subfamily 4, group A, member 3	−1.29	10^−4^	0.05
*21*	*Ghitm*	growth hormone inducible transmembrane protein	0.40	10^−4^	0.05
*22*	*Shox2*	short stature homeobox 2	6.18	10^−4^	0.05
*23*	*Insig1*	insulin induced gene 1	0.49	10^−4^	0.05
*24*	*Orai1*	ORAI calcium release-activated calcium modulator 1	1.83	10^−4^	0.05
*25*	*Thrsp*	thyroid hormone responsive	1.43	10^−4^	0.05
*26*	*Spint1*	serine peptidase inhibitor, Kunitz type 1	−0.91	10^−4^	0.05
*27*	*Liph*	lipase H	3.28	10^−4^	0.05
*28*	*Pla2g2c*	phospholipase A2, group IIC	−1.08	10^−4^	0.05
*29*	*Lilrb3l*	leukocyte immunoglobulin-like receptor, subfamily B, member 3-like	7.34	10^−4^	0.05
*30*	*Hspa1b*	heat shock protein family A (Hsp70) member 1B	−1.25	10^−4^	0.05
*31*	*Nmral1*	NmrA-like redox sensor 1	1.18	10^−4^	0.05
*32*	*Mogat2*	monoacylglycerol O-acyltransferase 2	2.08	10^−4^	0.05
*33*	*Defb17*	defensin β 17	6.02	10^−4^	0.05
*34*	*Sh3bgr*	SH3 domain binding glutamate-rich protein	1.11	10^−4^	0.05
*35*	*Eif2b3*	eukaryotic translation initiation factor 2B subunit γ	0.63	10^−4^	0.05
*36*	*Fcrl2*	Fc receptor-like 2	1.12	10^−4^	0.05
*37*	*Fuca1*	α-L-fucosidase 1	1.10	10^−4^	0.05
*38*	*Bdh1*	3-hydroxybutyrate dehydrogenase 1	0.37	10^−4^	0.05
*39*	*Rps16*	ribosomal protein S16	1.32	10^−3^	0.05
*40*	*Ifi27l2b*	interferon-α-inducible protein 27 like 2B	2.36	10^−3^	0.05
*41*	*Ifi47*	interferon-γ-inducible protein 47	1.47	10^−3^	0.05
*42*	*Mcm10*	minichromosome maintenance 10 replication initiation factor	−1.98	10^−3^	0.05
*43*	*Fjx1*	four-jointed box kinase 1	0.83	10^−3^	0.05
*44*	*Zmym6*	zinc finger MYM-type containing 6	−0.59	10^−3^	0.05
*45*	*Use1*	unconventional SNARE in the ER 1	1.11	10^−3^	0.05
*46*	*Fus*	FUS RNA-binding protein	0.48	10^−3^	0.05

Note: log2, see the legend of Figure 1; *p*, the statistical significance according to Fisher’s Z-test without the Benjamini correction for multiple comparisons.

**Table 5:** The qPCR data on the selected DEGs in the hypothalamus of the independently prepared eight other tame adult male rats and eight other aggressive ones (all unrelated animals).

Study Design			Behavioral “Glove” Test [20] and the qPCR Data on Gene Expression [This Work]								
Rat		No.	1	2	3	4	5	6	7	8	
	Set										
Glove Test	A		−3	−3	−3	−3	−3	−3	−3	−3	
	T		3	3	3	3	3	3	3	3	
*DEG*	Set		Relative Expression with Respect to Four Reference Genes, qPCR, M_0_ ± SEM								TOTAL
***Ascl3***	**A**		0.22 ± 0.04	0.14 ± 0.03	1.04 ± 0.12	0.11 ± 0.03	0.22 ± 0.04	0.14 ± 0.02	0.12 ± 0.03	0.12 ± 0.03	0.26 ± 0.12
	**T**		1.05 ± 0.28	1.35 ± 0.35	2.05 ± 0.11	1.95 ± 0.32	2.35 ± 0.24	2.61 ± 0.32	2.08 ± 0.61	2.86 ± 1.10	2.04 ± 0.52
***Apobec1***	**A**		1.83 ±0.19	0.71 ± 0.27	0.30 ± 0.08	0.58 ± 0.17	0.22 ± 0.09	0.58 ± 0.14	1.86 ± 0.28	0.93 ± 0.16	0.88 ± 0.28
	**T**		2.09 ± 0.62	9.22 ± 0.15	1.12 ± 0.05	1.12 ± 0.07	4.03 ± 0.73	3.83 ± 0.08	3.49 ± 1.13	0.45 ± 0.11	3.17 ± 1.07
***Defb17***	**A**		0.01 ± 0.01	0.01 ± 0.01	ND	ND	ND	0.01 ± 0.01	0.01 ± 0.01	0.01 ± 0.01	0.01 ± 0.01
	**T**		1.57 ± 0.22	1.70 ± 0.07	2.12 ± 0.51	1.20 ± 0.35	0.66 ± 0.04	1.51 ± 0.56	0.90 ± 0.04	0.99 ± 0.06	1.33 ± 0.35

Note. A, aggressive rats; T, tame rats; M_0_ ± SEM, mean ± standard error of the mean for three technical replicates; ND, not detected.

**Table 6:** The hypothalamic DEGs of tame versus aggressive rats found in this work in comparison with their known homologous DEGs in domestic animals versus their wild congeners as reported by others.

#	Hypothalamic DEGs, Tame vs. Aggressive Rats			DEGs Within the Tissues of the Domestic Animals versus Their Wild Congeners						
	DEG	log2	P_ADJ_	Tame/Domestic	Wild/Aggressive	Tissue	*DEG*	log2	P_ADJ_	[Ref]
i	ii	iii	iv	v	vi	vii	viii	ix	x	xi
*1*	*Banp*	−0.82	10^−3^	rabbits	rabbits	hippocampus	*Banp*	−1.39	0.05	[28]
*2*	*Banp*	−0.82	10^−3^	rabbits	rabbits	parietal-temporal cortex	*Banp*	−1.21	10^−2^	[28]
*3*	*Cd22*	2.85	10^−2^	dogs	wolves	blood	*Cd22*	2.34	0.05	[24]
*4*	*Cd22*	2.85	10^−2^	foxes	foxes	pituitary	*Cd22*	0.32	10^−2^	[23]
*5*	*Defb17*	6.02	0.05	rabbits	rabbits	parietal-temporal cortex	*Defb1*	1.19	10^−2^	[28]
*6*	*Eif2b3*	0.63	0.05	guinea pigs	cavy	frontal cortex	*Eif2b3*	0.72	10^−3^	[25]
*7*	*Fcgr3a*	2.06	10^−2^	rabbits	rabbits	parietal-temporal cortex	*Fcgr3b*	1.35	10^−2^	[28]
*8*	*Fcrl2*	1.12	0.05	foxes	foxes	pituitary	*Fcrl1*	0.43	10^−2^	[23]
*9*	*Ghitm*	0.40	0.05	guinea pigs	cavy	frontal cortex	*Ghitm*	−0.58	0.05	[25]
*10*	*Hbb-b1*	−3.97	10^−2^	dogs	wolves	blood	*Hbbl*	−5.92	10^−8^	[24]
*11*	*Hbb-b1*	−3.97	10^−2^	dogs	wolves	blood	*Hba1*	−4.06	10^−5^	[24]
*12*	*Hbb-b1*	−3.97	10^−2^	chicken	chicken	pituitary	*Hbad*	−1.07	10^−2^	[29]
*13*	*Hbb-b1*	−3.97	10^−2^	dogs	wolves	blood	*Hbm*	−6.46	10^−6^	[24]
*14*	*Hbb-b1*	−3.97	10^−2^	dogs	wolves	blood	*Hbz1*	−7.10	10^−2^	[24]
*15*	*Hspa1b*	−1.25	0.05	rabbits	rabbits	parietal-temporal cortex	*Hspa5*	−1.12	0.05	[28]
*16*	*Hspa1b*	−1.25	0.05	rabbits	rabbits	amygdala	*Hspa5*	−1.12	0.05	[28]
*17*	*Hspa1b*	−1.25	0.05	rabbits	rabbits	parietal-temporal cortex	*Hspa8*	−1.46	10^−9^	[28]
*18*	*Hspa1b*	−1.25	0.05	rabbits	rabbits	amygdala	*Hspa8*	−1.10	0.05	[28]
*19*	*Hspa1b*	−1.25	0.05	rabbits	rabbits	hippocampus	*Hspa8*	−1.36	10^−2^	[28]
*20*	*Ifi27l2b*	2.36	0.05	chicken	chicken	pituitary	*Ifi6*	−2.49	10^−4^	[29]
*21*	*Krt2*	−1.65	10^−4^	chicken	chicken	pituitary	*Krt17*	−1.12	0.05	[29]
*22*	*Liph*	3.28	0.05	guinea pigs	cavy	frontal cortex	*Lipa*	0.84	10^−2^	[25]
*23*	*Liph*	3.28	0.05	guinea pigs	cavy	frontal cortex	*Lipm*	1.45	10^−2^	[25]
*24*	*Liph*	3.28	0.05	chicken	chicken	pituitary	*Lipml*	0.55	10^−3^	[29]
*25*	*Mogat2*	2.08	0.05	rabbits	rabbits	hippocampus	*Mogat1*	−1.93	0.05	[28]
*26*	*Morn1*	1.24	10^−6^	foxes	foxes	pituitary	*Morn2*	−0.25	0.05	[23]
*27*	*Morn1*	1.24	10^−6^	guinea pigs	cavy	frontal cortex	*Morn2*	0.89	0.05	[25]
*28*	*Mre11a*	−2.47	10^−3^	guinea pigs	cavy	frontal cortex	*Mre11a*	1.42	10^−4^	[25]
*29*	*Nr4a3*	−1.29	10^−4^	chicken	chicken	pituitary	*Nr4a3*	−0.85	0.05	[29]
*30*	*Nr4a3*	−1.29	10^−4^	rabbits	rabbits	amygdala	*Nr4a3*	−1.58	0.05	[28]
*31*	*Nr4a3*	−1.29	10^−4^	guinea pigs	cavy	frontal cortex	*Nr2c1*	−0.74	10^−2^	[25]
*32*	*Nr4a3*	−1.29	10^−4^	chicken	chicken	pituitary	*Nr3c1*	0.51	10^−5^	[29]
*33*	*Nr4a3*	−1.29	10^−4^	guinea pigs	cavy	frontal cortex	*Nr5a1*	−2.19	0.05	[25]
*34*	*Orai1*	1.83	0.05	guinea pigs	cavy	frontal cortex	*Orai1*	−1.30	10^−3^	[25]
*35*	*P2rx4*	1.14	0.05	guinea pigs	cavy	frontal cortex	*P2rx6*	0.55	0.05	[25]
*36*	*Pcdhb9*	−1.01	0.05^4^	guinea pigs	cavy	frontal cortex	*Pcdh20*	−0.73	0.05	[25]
*37*	*Pcdhb9*	−1.01	0.05^4^	guinea pigs	cavy	frontal cortex	*Pcdhac1*	0.72	10^−2^	[25]
*38*	*Pcdhb9*	−1.01	0.05^4^	rabbits	rabbits	parietal-temporal cortex	*Pcdhb15*	−1.04	0.05	[28]
*39*	*Pcdhb9*	−1.01	0.05^4^	rats	rats	frontal cortex	*Pcdhga1*	2.10	10^−5^	[25]
*40*	*Pcdhb9*	−1.01	0.05^4^	rabbits	rabbits	amygdala	*Pcdhgb4*	1.53	10^−4^	[28]
*41*	*Pcdhb9*	−1.01	0.05^4^	rabbits	rabbits	parietal-temporal cortex	*Pcdhgb4*	1.06	10^−4^	[28]
*42*	*Pcdhb9*	−1.01	0.05^4^	rabbits	rabbits	hypothalamus	*Pcdhgb4*	1.67	10^−2^	[28]
*43*	*Pla2g2c*	−1.08	0.05	rabbits	rabbits	parietal-temporal cortex	*Pla1a*	1.35	10^−2^	[28]
*44*	*Pla2g2c*	−1.08	0.05	guinea pigs	cavy	frontal cortex	*Pla2g4a*	−1.74	10^−7^	[25]
*45*	*Pla2g2c*	−1.08	0.05	rabbits	rabbits	parietal-temporal cortex	*Pla2g4c*	2.29	10^−8^	[28]
*46*	*Pla2g2c*	−1.08	0.05	rabbits	rabbits	amygdala	*Pla2g4c*	2.34	10^−3^	[28]
*47*	*Pla2g2c*	−1.08	0.05	rabbits	rabbits	hippocampus	*Pla2g4c*	1.63	0.05	[28]
*48*	*Pla2g2c*	−1.08	0.05	guinea pigs	cavy	frontal cortex	*Pla2g5*	−1.01	0.05	[25]
*49*	*Pla2g2c*	−1.08	0.05	chicken	chicken	pituitary	*Pla2g7*	−0.83	10^−2^	[29]
*50*	*Rbm3*	1.04	10^−3^	guinea pigs	cavy	frontal cortex	*Rbm11*	1.02	0.05	[25]
*51*	*Sh3bgr*	1.11	0.05	guinea pigs	cavy	frontal cortex	*Sh3bgr*	0.99	10^−2^	[25]
*52*	*Shox2*	6.18	0.05	rabbits	rabbits	hippocampus	*Shox2*	−3.43	10^−3^	[28]
*53*	*Spint1*	−0.91	0.05	dogs	wolves	blood	*Spint1*	5.28	10^−2^	[24]
*54*	*Tpm2*	1.44	10^−2^	foxes	foxes	pituitary	*Tpm4*	0.84	10^−8^	[23]

**Table 7:** Correlations between the significant differential gene expression in the tame versus aggressive rats under study and significant differential gene expression in domestic animals versus their wild congers presented in Table 6.

	log2 Value	Signs of Log2 Values of Homologous DEGs		Binomial Distribution	χ^2^ Test		Fisher’s Exact Test
Homology Type		PC1: Same Signs	PC2: Opposite Signs		χ^2^	*p*	
i	ii	iii	iv	v	vi	vii	viii
**Number of Orthologous DEG Pairs**	**Many Homologs**	7	0	10^−3^	7.78	10^−2^	0.05
	**Only One Gene**	2	5	0.23			

**Table 9:** Correlations between the effects of co-directed changes in the expression of homologous genes on the human reproductive potential and on various traits during the divergence of the domestic and wild animals from their most recent common ancestor.

	Human	Effect of Gene Expression Changes on Human Reproductive Potential		Binomial Distribution	Pearson’s χ^2^ -Test		Fisher’s Exact Test
Animals		Decreased (→)	Increased (←)		χ^2^	*p*	
i	ii	iii	iv	v	vii	viii	ix
**Gene Expression Changes During Divergence from Most Recent Common Ancestor**	**domestic**	13	1	10^−3^	6.30	0.05	0.05
	**wild**	7	7	0.60			

## Data Availability

The primary RNA-Seq data obtained in this work were deposited in the NCBI SRA database (ID = PRJNA668014).

## Supplementary Materials

The following are available online at https://www.mdpi.com/article/10.3390/ani11092667/s1, Table S1. Statistically significant correlations between the relative expression levels of the seven differentially expressed genes (DEGs) and one reference genes within the hypothalamus of tame versus aggressive rats, which were measured experimentally in vivo using RNA-Seq [this work] and qPCR [34] methods. Table S2. Effects of underexpression or overexpression of the human genes under this study on the human diseases through aggressiveness changes, as estimated [56,57].
