# Supplementary material for: Domestication Explains Two-Thirds of Differential-Gene-Expression Variance between Domestic and Wild Animals; The Remaining One-Third Reflects Intraspecific and Interspecific Variation
Source: Animals (Basel). 2021 Sep 10;11(9):2667. doi: 10.3390/ani11092667 (PMC8465180; doi:10.3390/ani11092667)
Supplement: Supplementary file 1 [file animals-11-02667-s001.zip › 2)_Animals-1343638_Supplement_R0_Chadaeva(Ponomarenko).pdf]

animals

SUPPLEMENTARY MATERIALS

Domestication Explains Two-Thirds of Differen-tial-Gene-Expression Variance between Domestic and Wild Animals; the Remaining One-Third Reflects Intraspecific and Interspecific Variation

Irina Chadaeva, Petr Ponomarenko, Rimma Kozhemyakina, Valentin Suslov, Anton Bogomolov, Natalya Klimova, Svetlana Shikhevich, Ludmila Savinkova, Dmitry Oshchepkov, Nikolay Kolchanov, Arcady Markel, Mikhail Ponomarenko*

Institute of Cytology and Genetics, Siberian Branch of Russian Academy of Sciences, Novosibirsk 630090, Russia;
* Correspondence: pon@bionet.nsc.ru (M.P.)

Supplementary Results

Table S1. Statistically significant correlations between the relative expression levels of the seven differentially expressed genes (DEGs) and one reference genes within the hypothalamus of tame versus aggressive rats, which were measured experimentally *in vivo* using RNA-Seq [this work] and qPCR [1] methods

#	Rat gene		Differential expression, qPCR, six tame adult male rats vs six aggressive ones, 70 generations of the artificial selection for aggressiveness or tameness [1]			Differential expression, RNA-Seq, three tame adult male rats vs three aggressive ones, 90 generations of the artificial selection for aggressiveness or tameness [this work]		
	Symbol	**Name**	$P_{Mann–Whitney}$	P_Z	**$\log_2$**	**$\log_2$**	P_{Z-}	P_{ADJ}
**1**	Cacna2d3	calcium voltage-gated channel auxiliary subunit α2δ3	*0.05*	*0.05*	**−0,4**	**−0,14**	*0.60*	*0.79*
**2**	*Gad2*	glutamate decarboxylase 2	*10^{-2}*	*10^{-2}*	**−0,7**	**−0,61**	*0.29*	*0.60*
**3**	*Gria2*	glutamate ionotropic receptor ampa type subunit 2	*0.05*	*0.05*	**−0,2**	**−0,53**	*0.12*	*0.47*
**4**	*Mapk1*	mitogen-activated protein kinase 1	*0.05*	*0.05*	**−0,4**	**−0,06**	*0.57*	*0.78*
**5**	*Nos1*	nitric oxide synthase 1	*0.05*	*0.05*	**−0,5**	**−0,55**	*0.36*	*0.64*
**6**	*Pomc*	proopiomelanocortin	*0.05*	*0.05*	**−0,2**	**0,73**	*0.19*	*0.54*
**7**	*Syn1*	synapsin 1	*0.05*	*0.05*	**−0,3**	**−0,28**	*0.18*	*0.53*
**8**	*Rpl30*	ribosomal protein L30 (reference gene)	*0.05*	*0.05*	**0,0**	**0,64**	*0.59*	*0.87*
	**Pearson's linear correlation (the statistical significance estimate):**					**r = 0.71 ($p < 0.05$)**		

Notes: DEG differentially expressed genes ; $\log_2$, $\log_2$-transformed fold change (i.e., a ratio of a gene expression level in tame rats to that in aggressive rats); **$P_{Mann–Whitney}$**, the confidence levels of the statistical significance estimates according to Mann–Whitney U-test. P_Z and P_{ADJ}, the confidence levels of the statistical significance estimates according to Fisher's Z-test without and with Benjamini correction for multiple comparisons, respectively; the difference in the decisions made on the genes *Cacna2d3*, *Gad2*, *Gria2*, *Mapk1*, *Nos1*, *Pomc*, and *Syn1* as statistically significant [1] and insignificant [this work] DEGs of tame versus aggressive rats corresponds to difference in the total number of 12 [1] and 6 [this work] animals, which were used to make these decisions according to requirements for qPCR [1] and RNA-Seq [this work] measurement systems used; Rpl30, ribosomal protein L30 as a reference gene.

Table S2. Effects of underexpression or overexpression of the human genes under this study on the human diseases through aggressiveness changes, as estimated [2, 3].

#	Human Gene	Deficit (↓)			Excess (↑)		
		N_{SNP}	Effect on the human diseases through an aggressiveness change [Ref]	$	N_{SNP}	Effect on the human diseases through an aggressiveness change [Ref]	$
1	*ACKR1*	1 [2]	in human behavior models using Ackr1-null mice: impaired balance, high risks of anxiety, whole-body tremor and hypoactivity under stress [6]	→		within cohort-based study: ACKR1-excess contributes to mortality of men with coronary artery diseases [7]	→
2	*AKAP17A*	6 [3]	within a cohort-based study: predisposition to accelerated aging in men [8]	→	13 [3]	according to induced abortions due to fetal Klinefelter syndrome compared with those from spontaneous ones of normally developed male fetuses: increased risk of testicular degeneration [9]	→
3	*AMELY*	1 [3]	in line with post-mortem peripheral blood samples of male suicide completers in comparison with age-matched men of healthy living: increased risk of suicide in men [10]	→		based on clinical case-control studies of patients with diabetic foot ulcers (n=5), venous leg ulcers (n=5) and pyoderma gangrenosum (n=2): exogenous recombinant amelogenin is a wound healing drug [11]	←
4	*APOA1*	1 [2]	in accordance with a cohort-based study: higher risk of mental disorders according to low score of Montreal Cognitive Assessment (MoCA) [12]	→		as per a cohort-based immune-histochemical study of infertile women compared with fertile women: unexplained infertility in women [13]	→
5	*AR*		as reported by a retrospective clinical review: higher risk of early mortality through metabolic diseases because of disturbed gut microbiota [14]	→	3 [2]	in agreement with a clinical cohort-based cytological studies of androgenetic alopecia patients versus a norm men of the same age: androgen-induced premature aging in adult men [15]	→
6	*ASMT*	3 [3]	in conformity with human allergic airway inflammation models using mice: higher risks of inflammatory airway diseases such as asthma because of melatonin deficiency [16]	→	10 [3]	within a men reproductive health immunohistochemical model using ram's seminal plasma and reproductive tract samples: melatonin excess protects sperm from oxidative DNA damage [17]	←
7	*ASMTL*	5 [3]	as shown by a cohort-based study of prostatic hyperplasia tissue biopsies using quantitative polymerase chain reaction (qPCR): increased risk of prostate cancer [18]	→	13 [3]	in agreement with a cohort-based study of the sporadic autism patients who had additionally intellectual disability, craniofacial anomaly, or seizure: increased risk of autism spectrum disorders [19]	→
8	*CD99*	3 [3]	in a human atherogenesis model using mice administered with vector pcDNA3 carrying the *Cd99* gene fragment for extracellular domain: lesser risks of stroke and infarction as most often causes of human death [20]	←	20 [3]	within a human disease model using mice: both prevent and delayed progression of acute myeloid leukemia, as well as lowered leukemia engraftment in the bone marrow [21]	←
9	*CDY2A*	1 [3]	in keeping with a qPCR study of men with Y-chromosome microdeletions: male maturation arrest [22]	→		pursuant to qPCR, histological and cytological studies of the testicular tissue in men with an abnormal karyotype or a Y-chromosome microdeletion: partly repaired fertility in men due to the *CDY1* paralog [23]	←
10	*CETP*	1 [2]	according to the CETP promoter of a proband, who is a heterozygote of 18-bp deletion containing TATA box: atherogenesis delay reduces risks of both myocardial infarction and stroke, which are most often causes of human death [24]	←	3 [2]	as claimed by a cohort-based study: CETP excess during pregnancy elevates risk for later diabetes development [25]	→
11	*CRLF2*	2 [3]	within human respiratory disease models using Crlf2-knockout mice: weakened symptoms of acute respiratory tract infections in children and the elderly [26]	←	4 [3]	as reported by a cohort-based transcriptome profilling: higher mortality in pediatric acute lymphoblastic leukemia [27]	→

Note: N_{SNP}, as the number of candidate SNP markers that significantly decrease or increase the affinity of the TATA-binding protein (TBP) for the promoters of the considered gene according to estimates cited as [Ref] and, thereby, decrease (↓) or increase (↑) the expression of this gene, as has been repeatedly proven by many independent experiments (e.g., [4], for a review, see [5]); ***$***, as effects on the human reproductive potential changes: decrease (→) or increase (←). ***Genes***: *ACKR1*, atypical chemokine receptor 1 (synonym: Duffy blood group); *AKAP17A*, A-kinase anchoring protein 17A; *AMELY*, amelogenin Y-linked; *APOA1*, apolipoprotein A1; *AR*, androgen receptor; *ARTN*, artemin; *ASMT*, acetylserotonin O-methyltransferase; *ASMTL*, N-acetylserotonin O-methyltransferase-like protein; *CD99*, CD99 molecule (synonym: Xg blood group); *CDY2A*, chromodomain Y-linked 2A; *CETP*, cholesteryl ester transfer protein; *CRLF2*, cytokine receptor like factor 2;

Table S2. Cont.

#	Human Gene	Deficit (↓)			Excess (↑)		
		N_{SNP}	**Effect on the human diseases through an aggressiveness change [Ref]**	**$**	N_{SNP}	**Effect on the human diseases through an aggressiveness change [Ref]**	**$**
12	*CSF2RA*	9 [3]	as specified by human embryogenesis model using the bovine embryo, whose Csf2ra genes were disrupted using CRISPR/Cas9-system: impaired blastocyst development [28]	→	4 [3]	as maintained by human respiratory disease models using mice: lentiviral vectors carrying the mouse Csf2ra gene have passed preclinical trials in mice for the treatment of respiratory failure [29]	←
13	*CYP2A6*	2 [2]	as said by a cohort-based non-smoking pregnant women study: reduced damage from passive smoking for non-smoking pregnant women [30]	←		within human drug addiction models using mice:sons born from smoking mothers could be more susceptible to nicotine dependence later in life. [31]	→
14	*CYP2B6*	2 [2]	as observed by a cohort-based study: higher risks of autism spectrum disorders in offspring under maternal exposure to environmental pollutants [32]	→		as said by a cohort-based study: lesser serum concentrations of exogenous toxins, which dietary intake from polluted environment [33]	←
15	*CYP17A1*	1 [2]	within human steroidogenesis models using adult male rats: higher risks of male infertility through decreased testosterone levels and, thus, impaired testicular steroidogenesis [34]	→	1 [2]	according to alternative traditional Asiatic medicine: Malaysian propolis increases CYP17A1 level in the testes as a drug to overcome subfertility in diabetics [35]	←
16	*DHFR*	3 [2]	within a combined microbiological, cytological and pharmaceutical study: a synthetic DHFR-inhibitor seems to be a promising anti-mycobacterial drug against tuberculosis [36]	→	2 [2]	as known due to by a cohort-based Chinese Han population study on manifestation of SNP rs 11614913:T/T reinforcing the expression of DHFR: higher risks of recurrent spontaneous abortion [37]	→
17	*DHRSX*	6 [3]	within a human disease model using HeLa cells, DHRSX knockdown reduces autophagy level as response to starvation [38]	→	3 [3]	as learned due to by a cohort-based ischemic stroke men patients versus healthy men study: increased risk of ischemic stroke in men in middle age, who are at reproductive age [39]	→
18	*DNMT1*	2 [2]	as per a retrospective cohort-based studying demographic, laboratory, outcome and mutational data of myeloid malignancy patients: decitabine treats myeloid tumor via depleting epigenetic DNMT1 regulator [40]	←	7 [2]	within model of human disease using mice, increased risks of epigenetic disorders of fetal brain development under stress [41]	→
19	*ESR2*	2 [2]	within model of human disease using rats, ESR2-deficiency in adolescents reduces sperm quality in adults [42]	→		within model of human disease using rats, ESR2-excess in adolescents reduces sperm quality in adults [42]	→
20	*F2*		as said by a biomedical heuristic hypothesis based on available limited clinical data on new COVID-19 infection: α1-antitrypsin inhibits F2 and, thus, prevents micro- and macrothrombosis in order to relieve COVID-19 [43]	←	2 [2]	within a cytological study using cell line of extravillous trophoblasts of first trimester: increased risks of preeclampsia as one of the most challenging problems of modern obstetrics [44]	→
21	*F3*	2 [2]	as resulted of a retrospective cohort-based studying gender, age, laboratory, and treatment data of patients: ozone therapy suppresses F3 and, thereby, prevents thrombotic ischemic intestinal damage [45]	←	5 [2]	as reported by a retrospective clinical data review: increased risks of preeclampsia as one of the most challenging problems of modern obstetrics [46]	→
22	*F7*	2 [2]	as stated in a cohort-based study: increased risks of episodic spontaneous difficult to stop life-threatening bleeding [47]	→	5 [2]	according to a clinical case-report: exogenous recombinant activated F7 is a life-saving drug for obstetric life-threatening bleeding [48]	←
23	*F8*		according to a retrospective clinical review on 25 years of experience of diagnosis in hemophilia in the Mexican population: spontaneous hemorrhages in the brain, joints, muscles, internal organs and disability [49]	→	1 [2]	in agreement with a cohort-based study: increased risks of thrombosis provoking stroke and myocardial infarction as the two most frequent causes of death in humans [50]	→

Genes: *CSF2RA*, colony stimulating factor 2 receptor subunit α; *CYP17A1*, steroid 17α-monooxygenase; *CYP2A6*, xenobiotic monooxygenase; *CYP2B6*, 1,4-cineole 2-exo-monooxygenase; *DHFR*, dihydrofolate reductase; *DHRSX*, *d*ehydrogenase/reductase X-linked; *DNMT1*, DNA methyltransferase 1; *ESR2*, estrogen receptor 2 (β); *F2, F3, F7, and F8*, coagulation factors II (synonym: thrombin), III (synonyms: thromboplastin, tissue factor), VII (synonym: proconvertin), VIII (synonym: hemophilia A), respectively;

Table S2. Cont.

#	Human Gene	Deficit (↓)			Excess (↑)		
		N_{SNP}	Effect on the human diseases through an aggressiveness change [Ref]	$	N_{SNP}	Effect on the human diseases through an aggressiveness change [Ref]	$
24	*F9*	1 [2]	as observed by human hemophilia B models using mice: spontaneous hemorrhages in the brain, joints, muscles, internal organs and, eventually, disability [51]	→	1 [2]	s detected by human disease models using transgenic mice: increased risks of myocardial fibrosis causing tachyarrhythmias, disability *via* heart failure and, ultimately, cardiovascular death [52]	→
25	*F11*	1 [2]	as published within a clinical case-report: coagulation factor XI insufficiency provoking spontaneous bleeding and, ultimately, disability [53]	→	5 [2]	in relation to a cohort-based study: increased risks of spontaneous miscarriage [54]	→
26	*GCG*	2 [2]	as known due to human disease models using Gcg-knockout mice: higher risks of diabetic polyneuropathy development [55]	→		as met within human reproductive health models using dairy goats: reduced pregnancy rate [56]	→
27	*GH1*	2 [2]	as reported by a retrospective clinical review on 30 years of experience somatotropin replacement therapy in adult: increased mortality from cardiovascular disease [57]	→	2 [2]	as detected within a cohort-based study of women undergoing infertility treatment with somatotropin compared to without it: somatotropin prolongs the reproductive age in women [58]	←
28	*GJA5*	3 [2]	as learned within human cardiovascular disease models using Gja5-knockout mice: increased risks of the heart morphogenesis disorders, which result in arrhythmias and cardiovascular diseases [59]	→		as shown by cytological study using wild-typed mouse embryonic stem cells: increased arteriogenesis as the human body response to a low oxygen level at chronic hypoxia [60]	←
29	*GSTM3*	2 [2]	as revealed within a cohort-based study of men with small versus normal testicular volume: increased risk of non-obstructive azoospermia [61]	→	2 [2]	within human diseases model using cows, increased frequency of natural fertilization compared to artificial fertilization [62]	←
30	*GTPBP6*	3 [3]	increased intelligence quotient IQ scores in men [63] that is negatively significantly associated with amount of their siblings and cousins [64]	←	3 [3]	reduced intelligence quotient IQ scores in men [63] that is positively significantly associated with amount of their siblings and cousins [64]	→
31	*HBB*	9 [2]	hemoglobin deficit (thalassemia) elevates rise risks of auto-aggressive impulsiveness up to suicide [65], women subfertility [66], under-threshold IQ and severe anxiety in children [67]	→		in cohort studies: elite athletes do high-altitude trains rising hemoglobin level before low-altitude matches that rises their win chances [68]	←
32	*HBD*	2 [2]	hemoglobin deficit (thalassemia) elevates rise risks of auto-aggressive impulsiveness up to suicide [65], women subfertility [66], under-threshold IQ and severe anxiety in children [67]	→		in cohort studies: elite athletes do high-altitude trains rising hemoglobin level before low-altitude matches that rises their win chances [68]	←
33	*HBG2*	1 [2]	hemoglobin deficit (thalassemia) elevates rise risks of auto-aggressive impulsiveness up to suicide [65], women subfertility [66], under-threshold IQ and severe anxiety in children [67]	→		in cohort studies: elite athletes do high-altitude trains rising hemoglobin level before low-altitude matches that rises their win chances [68]	←
34	*HSD17B1*	3 [2]	within human disease model using Hsd17b1-knockdown mice: suppression of hormone-dependent breast tumor growth [69]	←	1 [2]	as reported by a retrospective clinical study review: increased risk of breast cancer [70]	→
35	*IL1B*	1 [2]	as observed due to human disease model using transgenic mice: reduced risks of bone marrow hyperplasia and bone deformation in case of bacterial invasion [71]	←	1 [2]	as learned by a human chronopathology model using cultured primary human fibroblasts: increased circadian hypersensitivity to pain [72]	→
36	*IL3RA*	2 [3]	within a human cancer model using acute myeloid leukemia cells, SS30 thioaptamer inhibites IL3RA that increases survival [73]	←	3 [3]	as detected due to a cohort-based study using microarrays: increased risks of acute myeloid leukemia in children [74]	→

Genes: *F9 and F11*, coagulation factors IX (synonym: hemophilia B) and XI, respectively; *GCG*, glucagon *GH1*, growth hormone 1 (synonym: somatotropin); *GJA5*, connexin 40 (synonym: gap junction protein α5); *GSTM3*, glutathione S-transferase μ3; *GTPBP6*, GTP-binding protein 6; *HBB*, *HBD*, and *HBG2*, hemoglobin subunits β, δ, and γ2, respectively; *HSD17B1*, hydroxysteroid 17β dehydrogenase 1; *IL1B*, interleukin 1β; *IL3RA*, interleukin 3 receptor subunit α..

Table S2. Cont.

#	Human Gene	Deficit (↓)			Excess (↑)		
		N_{SNP}	Effect on the human diseases through an aggressiveness change [Ref]	$	N_{SNP}	Effect on the human diseases through an aggressiveness change [Ref]	$
37	*IL9R*	1 [3]	as observed by a cohort-based study using quantitative polymerase chain reaction (qPCR): trophoblast implantation impaired within preeclampsia [75]	→	1 [3]	as revealed within human disease models using Il9r-knockout mice: increased risks of life-threatening anaphylactic shock [76]	→
38	*INS*	1 [2]	within a model of human diseases using sheeps, hypoinsulinemia slows down fetal growth and development [77]	→	2 [2]	as deposited with the ClinVar database: increased risks of neonatal diabetes mellitus, which can often progress to type I diabetes mellitus [78]	→
39	*KDM5D*	3 [3]	as resulted within a cytogenetic study using quantitative polymerase chain reaction (qPCR): increased risks of aggressive prostate cancer [79]	→		in line with human disease models using mice proteome: increased risks of cardiovascular diseases [80]	→
40	*LEP*	1 [2]	as per a biochemical study of women, who had had secondary hypothalamic amenorrhea: higher risks of this disease with dysfunction of hypothalamus endocrine axes and, ultimately, subfertility [81]	→	2 [2]	as summarized within a retrospective nutritional biochemistry review: increased risks of subfertility as an obesity complication [82]	→
41	*MBL2*	2 [2]	within human models disease using Mbl2-knockout mice: relief of suffering when brain trauma [83]	←	1 [2]	as generalized due to a retrospective review on current therapeutic strategies against COVID-19: exogenous recombinant human MBL2 is used within adjuvant therapy against COVID-19 [84]	←
42	*MMP12*	2 [2]	within models of human diseases using MMP12-knockout mice, low differentiation of oligodendrocytes of the central nervous system [85]	→		according to a cohort-based study of primary trophoblasts isolated from placenta during elective vaginal terminations of first-trimester pregnancies: trophoblast implantation improved within pregnancy [86]	←
43	*MTHFR*	2 [2]	as summed up within a retrospective review on in vitro fertilizations for women with premature ovarian insufficiency interested for pregnancy : higher risks of thrombophilia [87]	→	4 [2]	in line with a cohort-based study of plasma and placenta from pregnant women: increased risks of preeclampsia as one of the most challenging problems of modern obstetrics [88]	→
44	*NLGN4Y*	1 [3]	as revealed due to a cohort-based study using quantitative polymerase chain reaction (qPCR): increased risks of both primary prostate cancer and its biochemically-induced recurrence [89]	→	2 [3]	as stated by a cohort-based infertile versus fertile women study using RNA-Seq with respect to male foetal microchimerism: increased risks of infertility [90]	→
45	*NOS2*		within human models disease using Nos2-nullt rats: higher risks of human schistosomiasis, caused by *Schistosoma species*, is a major public health problem affecting more than 700 million people in 78 countries [91]	→	1 [2]	as stated by a cohort-based comparative study of pregnant women with and without gestational diabetes mellitus: increased risks of diabetes mellitus in pregnancy as pre-diabetes of both type I and II [92]	→
46	*NR5A1*		in human disease models using Nr5a1-null male mice: hyper-anxiety in impaired aggressive sexual behavior up to male infertility in line with men patients carrying NR5A1-defects [93] as well as NR5A1 deficit can cause hypoestrogenism [94] leading to 1% female infertility [95]	→	4 [2]	a retrospective meta-analysis of PubMed content: NR5A1-excess causes the excessive estrogen biosynthesis rising risks of estrogen-dependent inflammatory disorders in women [96] and *vice versa* for men [97]	←
47	*P2RY8*	2 [3]	according to a cohort-based cytogenetical study: increased risks of acute lymphoblastic leukemia in children [98]	→	2 [3]	within a cohort-based study using mononuclear cells isolated from bone marrow aspirates of individuals with leukemia and conventionally healthy volunteers: increased risk of pediatric acute leukemia [99]	→
48	*PGR*	1 [2]	within a model of human diseases using PGR-knockout mice: infertility through embryo attachment impaired [100]	→	1 [2]	as claimed by a bioinformatics comparative analysis of microarray datasets downloaded from the GEO database: improved relapse-free survival after an estrogen receptor positive breast cancer recovery [101]	←

Genes: *IL9R*, interleukin 9 receptor *INS*, insulin; *KDM5D*, lysine demethylase 5D; *LEP*, leptin; *MBL2*, mannose binding lectin 2. *MMP12*, matrix metallopeptidase 12 (synonym: macrophage elastase); *MTHFR*, methylenetetrahydrofolate reductase; *NLGN4Y*, neuroligin 4 Y-linked; *NOS2*, nitric oxide synthase (inducible, hepatocytes, macrophage); ; *NR5A1*, steroidogenic factor 1; *P2RY8*, G-protein coupled purinergic P2Y receptor 8; *PGR*, progesterone receptor;

Table S2. Cont.

#	Human Gene	Deficit (↓)			Excess (↑)		
		N_{SNP}	Effect on the human diseases through an aggressiveness change [Ref]	$	N_{SNP}	Effect on the human diseases through an aggressiveness change [Ref]	$
49	*PLCXD1*	15 [3]	as maintained by a cohort-based study: increased risks of ischemic stroke and its complications in men of middle (reproductive) age [102]	→	35 [3]	within human cancer models using melanoma cells, transfection of a vector with PLCXD1 gene cDNA inhibits their proliferation [103]	←
50	*PPP2R3B*	3 [3]	within a model of human diseases using endemic for China carp fish *Gobiocypris rarus*, impaired spermatogenesis [104]	→	15 [3]	within human cancer models using melanoma cells, transfection of a plasmid with the PPP2R3B gene cDNA inhibits their growth [#5ob]	←
51	*PROC*	2 [2]	as generalized within a retrospective review: increased risks of life-threatening fulminant purpura in newborns [106]	→	6 [2]	within a model of human diseases using mice, increased risks of premature pregnancy loss [107]	→
52	*RPS4Y2*	1 [3]	as observed by a cohort-based study using transcriptome profiling: increased risks of male infertility [108]	→		as per a cohort-based study using quantitative polymerase chain reaction (qPCR) and microarray profiling: increased risks of metabolic fatty liver diseases leading to liver cirrhosis and eventually cancer [#109]	→
53	*SHOX*	5 [3]	in cohort-based studies: low SHOX causes short stature [110] as an adaptive epigenetic response to adverse life conditions, when each calorie saved due to short stature exalts fighting stress [111]	←	3 [3]	in cohort studies: girls carrying one extra SHOX copy have tall stature (without any other differences from a norm) [112], which elevates risks of pregnancy complications in military active-duty women [113]	→
54	*SLC25A6*	1 [3]	as summed up within a retrospective disease-ralated review on the human SLC25 gene family: increased risks of muscular dystrophy [114]	→	4 [3]	on the basis of human disease models using dual-luciferase reporter assay: increased resistance to human herpesvirus type 5, which increases morbidity and mortality with weakened immunity [115]	←
55	*SOD1*	1 [2]	within human disease models using Sod1-knockout male mice: decreased sperm motility and fertility *in vivo* [116]	→		as reported by a retrospective clinical review: increased both the bioavailability of copper in the germ cells and their protection against copper toxicity and oxidative stress [117]	←
56	*SPRY3*		as identified dwithin a cohort-based study: enhanced angiogenesis in tumors and cancer [118]	→	10 [3]	in consonance with human disease models using mice: gender-specifically increased risks of autism among men compared to women [119]	→
57	*STAR*	1 [2]	on the authority of human disease models using Star-knockout mice: increased risks of lipoid congenital adrenal hyperplasia [120]	→		as detected by a cohort-based study using quantitative polymerase chain reaction (qPCR): higher risks of primary adrenal tumors [121]	→
58	*TBL1Y*		as known due to a cohort-based study using quantitative polymerase chain reaction (qPCR): increased risks of violations of both cardiogenesis and heart rate in men [122]	←	2 [3]	according to a cohort-based study using quantitative polymerase chain reaction (qPCR): decreased risks of violations of both cardiogenesis and heart rate in men [122]	→
59	*THBD*	1 [2]	in harmony with human disease models using Thbd-knockout embryos mice: increased risks of placental insufficiency and fetal loss [123]	→		s said by a retrospective case-control study review: exogenous recombinant soluble human thrombomodulin is widely used as a drug against disseminated intravascular blood coagulation [124]	→
60	*TMSB4Y*		as learned due to a cohort-based study using quantitative polymerase chain reaction (qPCR): increased risks of prostate cancer [125]	→	1 [3]	in obedience to human disease models using male breast cancer samples: gender-specific improved tumor suppression in men [126]	←
61	*TPI1*	2 [2]	in compliance with human aging models using mice: higher risks of neurodegenerative disorders [127]	→		as specified by a cohort-based study using proteomics analysis: increased risks of intrahepatic cholangiocarcinoma as the second most common primary tumor leading to liver cancer [128]	→

Genes:; *PLCXD1*, phosphatidylinositol-specific phospholipase C, X domain containing 1; *PPP2R3B*, protein phosphatase 2 regulatory subunit β''β; *PROC*, protein C (synonym: inactivator of coagulation factors Va and VIIIa); *RPS4Y2*, ribosomal protein S4 Y-linked 2; *SHOX*, short stature homeobox; *SLC25A6*, adenine nucleotide translocator 3; *SOD1*, superoxide dismutase 1; *SPRY3*, sprouty RTK signaling antagonist 3; *STAR*, steroidogenic acute regulatory protein;. *TBL1Y*, transducin β like 1 Y-linked;. *THBD*, thrombomodulin; *TMSB4Y*, thymosin β4 Y-linked; *TPI1*, triosephosphate isomerase 1.

Table S2. Cont.

#	Human Gene	Deficit (↓)			Excess (↑)		
		N_{SNP}	Effect on the human diseases through an aggressiveness change [Ref]	$	N_{SNP}	Effect on the human diseases through an aggressiveness change [Ref]	$
62	*TSPY2*	1 [3]	as obtained by a cohort-based study increased risks of male infertility [129]	→	2 [3]	according to a cohort-based study: increased risks of testicular maturation arrest [129]	→
63	*TSPY4*		as detected by a cohort-based study using RNA-seq analysis: increased risks of spermatogenesis disorders [130]	→	1 [3]	as per a cohort-based study using RNA-seq analysis: a synthetic agonist of gonadotropin-releasing hormone as a drug for male infertility increases TSPY4 level [130]	←
64	*USP9Y*		as concluded by a retrospective review on mutations within both X and Y chromosomes: higher risks of spermatogenic dysfunction [131]	→	1 [3]	in line with a cohort-based comparative study of male compared with female patients with idiopathic dilated cardiomyopathy using microarray analysis: increased risk of de novo heart failure in men [132]	→
65	*UTY*	1 [3]	in agreement with human disease models using hemizygous Uty-knockout mice: increased risks of developmental defects in male embryos [133]	→		based on a comparative female versus male human neural stem Immunocytochemical analysis: gender-specifically improve neurogenesis within the treatment of the nervous system in men [134]	←
66	*VAMP7*	4 [3]	according to a bioinformatics analysis of datasets downloaded from The Cancer Genome Atlas (TCGA) database: increased overall survival of patients with esophageal adenocarcinoma [135]	←	9 [3]	in accordance with human disease models using transgenic mice: increased risks of subfertility [136]	→
67	*ZBED1*	1 [3]	as known due to a cytological study using quantitative polymerase chain reaction (qPCR): increased risks of subfertility through adenovirus excess within spermatozoa in the later stages of infection [137]	→	11 [3]	As detected by a cytological study using quantitative polymerase chain reaction (qPCR): increased risks of subfertility through adenovirus excess within spermatozoa in the early stages of infection [137]	→
68	*ZFY*		within a model of human diseases using bulls: subfertility through reduced spermatozoa motility [138]	→	2 [3]	increased risks spermatocyte meiosis arrests leading to their apoptosis, azoospermia and, ultimately, infertility [139]	→

Genes: *TSPY2* and *TSPY4*, testis specific protein Y-linked 2 and 4, respectively; *USP9Y*, ubiquitin specific peptidase 9 Y-linked; *UTY*, ubiquitously transcribed tetratricopeptide repeat containing, histone demethylase UTY Y-linked; *VAMP7*, vesicle associated membrane protein 7 (synonym: synaptobrevin-like protein 1); *ZBED1*, DNA replication-related element binding factor; *ZFY*, Zinc-finger protein Y-linked.

References

1. Oshchepkov, D., Ponomarenko, M., Klimova, N., Chadaeva, I., Bragin, A., Sharypova, E., Shikhevich, S., Kozhemyakina, R. A rat model of human behavior provides evidence of natural selection against underexpression of aggressiveness-related genes in humans. *Front Genet*. **2019**; *10*, 1267
2. Chadaeva, I., Ponomarenko, P., Rasskazov, D., Sharypova, E., Kashina, E., Zhechev, D., Drachkova, I., Arkova, O., Savinkova, L., Ponomarenko, M. et al. Candidate SNP markers of reproductive potential are predicted by a significant change in the affinity of TATA-binding protein for human gene promoters. *BMC Genomics*. **2018**; *19*, 0.
3. Ponomarenko, M., Kleshchev, M., Ponomarenko, P., Chadaeva, I., Sharypova, E., Rasskazov, D., Kolmykov, S., Drachkova, I., Vasiliev, G., Gutorova, N., *et al*. Disruptive natural selection by male reproductive potential prevents underexpression of protein-coding genes on the human Y chromosome as a self-domestication syndrome. *BMC Genetics*. **2020**; *21*, 89.
4. Mogno, I., Vallania, F., Mitra, R.D., Cohen, B. TATA is a modular component of synthetic promoters. *Genome Res*. **2010**; *20*, 1391-1397.
5. Ponomarenko, P., Suslov, V., Savinkova, L., Ponomarenko, M., Kolchanov, N. A precise equilibrium equation for four steps of binding between TBP and TATA-box allows for the prediction of phenotypical expression upon mutation. *Biofizika (Mosk)*. **2010**; *55*, 358-369.
6. Schneider, E.H., Fowler, S.C., Lionakis, M.S., Swamydas, M., Holmes, G., Diaz, V., Munasinghe, J., Peiper, S.C., Gao, J.L., Murphy, P.M. Regulation of motor function and behavior by atypical chemokine receptor 1. *Behav Genet*. **2014**; *44*, 498-515.
7. Hernandez-Aguilera, A., Fibla, M., Cabre, N., Luciano-Mateo, F., Camps, J., Fernandez-Arroyo, S., Martin-Paredero, V., Menendez, J.A., Sirvent, J.J., Joven, J. Chemokine (C-C motif) ligand 2 and coronary artery disease: tissue expression of functional and atypical receptors. *Cytokine*. **2020**; *126*, 154923

8. Lee, B.P., Pilling, L.C., Bandinelli, S., Ferrucci, L., Melzer, D., Harries, L.W. The transcript expression levels of HNRNPM, HNRNPA0 and AKAP17A splicing factors may be predictively associated with ageing phenotypes in human peripheral blood. *Biogerontology*. **2019**; *20*, 649-663.
9. Winge, S.B., Dalgaard, M.D., Jensen, J.M., Graem, N., Schierup, M.H., Juul, A., Rajpert-De, Meyts E., Almstrup, K. Transcriptome profiling of fetal Klinefelter testis tissue reveals a possible involvement of long non-coding RNAs in gonocyte maturation. *Hum Mol Genet*. **2018**; *27*, 430-439.
10. Kimura, A., Hishimoto, A., Otsuka, I., Okazaki, S., Boku, S., Horai, T., Izumi, T., Takahashi, M., Ueno, Y., Shirakawa, O., Sora, I. Loss of chromosome Y in blood, but not in brain, of suicide completers. *PLoS One*. **2018**; *13*, e0190667.
11. Chadwick, P., Acton, C. The use of amelogenin protein in the treatment of hard-to-heal wounds. *Br J Nurs*. **2009**; *18*, S22, S24, S26, passim.
12. Peng, Y., Zhou, L., Cao, Y., Chen, P., Chen, Y., Zong, D., Ouyang, R. Relation between serum leptin levels, lipid profiles and neurocognitive deficits in Chinese OSAHS patients. *Int J Neurosci*. **2017**; *127*, 981-987.
13. Manohar, M., Khan, H., Sirohi, V.K., Das, V., Agarwal, A., Pandey, A., Siddiqui, W.A., Dwivedi, A. Alteration in endometrial proteins during early- and mid-secretory phases of the cycle in women with unexplained infertility. *PLoS One*. **2014**; *9*, e111687.
14. Harada, N., Minami, Y., Hanada, K., Hanaoka, R., Kobayashi, Y., Izawa, T., Sato, T., Kato, S., Inui, H., Yamaji, R. Relationship between gut environment, feces-to-food ratio, and androgen deficiency-induced metabolic disorders. *Gut Microbes*. **2020**; *12*, 1817719.
15. Yang, Y.C., Fu, H.C., Wu, C.Y., Wei, K.T., Huang, K.E., Kang, H.Y. Androgen receptor accelerates premature senescence of human dermal papilla cells in association with DNA damage. *PLoS One*. **2013**; *8*, e79434.
16. Wu, H.M., Zhao, C.C., Xie, Q.M., Xu, J., Fei, G.H. TLR2-melatonin feedback loop regulates the activation of NLRP3 inflammasome in murine allergic airway inflammation. *Front Immunol*. **2020**; 11, 172.
17. Gonzalez-Arto, M., Hamilton, T.R., Gallego, M., Gaspar-Torrubia, E., Aguilar, D., Serrano-Blesa, E., Abecia, J.A., Perez-Pe, R., Muino-Blanco, T., Cebrian-Perez, J.A., Casao, A. Evidence of melatonin synthesis in the ram reproductive tract. *Andrology*. **2016**; *4*, 163-171.
18. Lau, Y.F., Zhang, J. Expression analysis of thirty one Y chromosome genes in human prostate cancer. *Mol Carcinog*. **2000**; *27*, 308-321.
19. Firouzabadi, S.G., Kariminejad, R., Vameghi, R., Darvish, H., Ghaedi, H., Banihashemi, S., Firouzkouhi Moghaddam, M., Jamali, P., Mofidi Tehrani, H.F., Dehghani, H., *et al*. Copy number variants in patients with autism and additional clinical features: report of VIPR2 duplication and a novel microduplication syndrome. *Mol Neurobiol*. **2017**; *54*, 7019-7027.
20. van Wanrooij, E.J., de Vos, P., Bixel, M.G., Vestweber, D., van Berkel, T.J., Kuiper J. Vaccination against CD99 inhibits atherogenesis in low-density lipoprotein receptor-deficient mice. *Cardiovasc Res*. **2008**; *78*, 590-596.
21. Vaikari, V.P., Du, Y., Wu, S., Zhang, T., Metzeler, K., Batcha, A.M.N., Herold, T., Hiddemann, W., Akhtari, M., Alachkar, H. Clinical and preclinical characterization of CD99 isoforms in acute myeloid leukemia. *Haematologica*. **2020**; *105*, 999-1012.
22. Stahl, P.J., Mielnik, A.N., Barbieri, C.E., Schlegel, P.N., Paduch, D.A. Deletion or underexpression of the Y-chromosome genes CDY2 and HSFY is associated with maturation arrest in American men with nonobstructive azoospermia. *Asian J Androl*. **2012**; *14*, 676-682.
23. Kleiman, S.E., Yogev, L., Hauser, R., Botchan, A., Bar-Shira, Maymon, B., Schreiber, L., Paz, G., Yavetz, H. Members of the CDY family have different expression patterns: CDY1 transcripts have the best correlation with complete spermatogenesis. *Hum Genet*. **2003**; *113*, 486-492.
24. Plengpanich, W., Le Goff, W., Poolsuk, S., Julia, Z., Guerin, M., Khovidhunkit, W. CETP deficiency due to a novel mutation in the CETP gene promoter and its effect on cholesterol efflux and selective uptake into hepatocytes. *Atherosclerosis*. **2011**; *216*, 370-373.
25. Ueland, T., Roland, M.C.P., Michelsen, A.E., Godang, K., Aukrust, P., Henriksen, T., Bollerslev, J., Lekva, T. Elevated cholesteryl ester transfer protein activity early in pregnancy predicts prediabetes 5 years later. *J Clin Endocrinol Metab*. **2020**; *105*, dgz119
26. Lay, M.K., Cespedes, P.F., Palavecino, C.E., Leon, M.A., Diaz, R.A., Salazar, F.J., Mendez, G.P., Bueno, S.M., Kalergis, A.M. Human metapneumovirus infection activates the TSLP pathway that drives excessive pulmonary inflammation and viral replication in mice. *Eur J Immunol*. **2015**; *45*, 1680-1695.
27. Tomar, A.K., Agarwal, R., Kundu, B. Most variable genes and transcription factors in acute lymphoblastic leukemia patients. *Interdiscip Sci*. **2019**; *11*, 668-678.
28. Xiao, Y., Uh, K., Negron-Perez, V.M., Haines, H., Lee, K., Hansen, P.J. Regulation of gene expression in the bovine blastocyst by colony-stimulating factor 2 is disrupted by CRISPR/Cas9-mediated deletion of CSF2RA. *Biol Reprod*. **2021**; *104*, 995-1007.
29. Arumugam, P., Suzuki, T., Shima, K., McCarthy, C., Sallese, A., Wessendarp, M., Ma, Y., Meyer, J., Black, D., Chalk, C., *et al*. Long-term safety and efficacy of gene-pulmonary macrophage transplantation therapy of PAP in Csf2ra-/- mice. *Mol Ther*. **2019**; *27*, 1597-1611.
30. Xie, C., Wen, X., Ding, P., Liu, T., He, Y., Niu, Z., Lin, J., Yuan, S., Guo, X., Jia, D., Chen W. Influence of CYP2A6*4 genotypes on maternal serum cotinine among Chinese nonsmoking pregnant women. *Nicotine Tob Res*. **2014**; *16*, 406-412.

31. Lkhagvadorj, K., Meyer, K.F., Verweij, L.P., Kooistra, W., Reinders-Luinge, M., Dijkhuizen, H.W., de Graaf, I.A.M., Plosch, T., Hylkema, M.N. Prenatal smoke exposure induces persistent Cyp2a5 methylation and increases nicotine metabolism in the liver of neonatal and adult male offspring. *Epigenetics*. **2020**; *15*, 1370-1385..
32. Traglia, M., Croen, L.A., Lyall, K., Windham, G.C., Kharrazi, M., DeLorenze, G.N., Torres, A.R., Weiss, L.A. Independent maternal and fetal genetic effects on midgestational circulating levels of environmental pollutants. *G3 (Bethesda)*. **2017**; *7*, 1287-1299.
33. Li, S., Shao, W., Wang, C., Wang, L., Xia, R., Yao, S., Du, M., Ji, X., Chu, H., Zhang, Z.,et al. Identification of common genetic variants associated with serum concentrations of p, p'-DDE in non-occupational populations in eastern China. *Environ Int*. **2021**; *152*, 106507
34. Milosevic, A., Bjelobaba, I., Bozic, I.D., Lavrnja, I., Savic, D., Tesovic, K., Jakovljevic, M., Stojilkovic, S.S., Janjic, M.M. Testicular steroidogenesis is suppressed during experimental autoimmune encephalomyelitis in rats. *Sci Rep*. **2021**; *11*, 8996.
35. Nna, V.U., Bakar, A.B.A., Ahmad, A., Umar, U.Z., Suleiman, J.B., Zakaria, Z., Othman, Z.A., Mohamed, M. Malaysian propolis and metformin mitigate subfertility in streptozotocin-induced diabetic male rats by targeting steroidogenesis, testicular lactate transport, spermatogenesis and mating behaviour. *Andrology*. **2020**; *8*, 731-746.
36. Hajian, B., Scocchera, E., Shoen, C., Krucinska, J., Viswanathan, K., G-Dayanandan, N., Erlandsen, H., Estrada, A., Mikusova, K., Kordulakova, J., *et al*. Drugging the folate pathway in mycobacterium tuberculosis: the role of multi-targeting agents. *Cell Chem Biol*. **2019**; *26*, 781-791.e6.
37. Wang, X., Zhang, L., Guan, C., Dong, Y., Liu, H., Ma, X., Xia, H. The polymorphism of rs11614913 T/T in pri-miR-196a-2 alters the miRNA expression and associates with recurrent spontaneous abortion in a Han-Chinese population. *Am J Transl Res*. **2020**; *12*, 1928-1941.
38. Zhang, G., Luo, Y., Li, G., Wang, L., Na, D., Wu, X., Zhang, Y., Mo, X., Wang, L. DHRSX, a novel non-classical secretory protein associated with starvation induced autophagy. *Int J Med Sci*. **2014**; *11*, 962-970.
39. Tian, Y., Stamova, B., Jickling, G.C., Xu, H., Liu, D., Ander, B.P., Bushnell, C., Zhan, X., Turner, R.J., Davis, R.R., *et al*. Y chromosome gene expression in the blood of male patients with ischemic stroke compared with male controls. *Gend Med*. **2012**; *9*, 68-75.e3.
40. Awada, H., Mahfouz, R.Z., Kishtagari, A., Kuzmanovic, T., Durrani, J., Kerr, C.M., Patel, B.J., Visconte, V., Radivoyevitch, T., Lichtin, A., *et al*. Extended experience with a non-cytotoxic DNMT1-targeting regimen of decitabine to treat myeloid malignancies. *Br J Haematol*. **2020**; *188*, 924-929.
41. Matrisciano, F,. Tueting, P., Dalal, I., Kadriu, B., Grayson, D.R., Davis, J.M., Nicoletti, F., Guidotti, A. Epigenetic modifications of GABAergic interneurons are associated with the schizophrenia-like phenotype induced by prenatal stress in mice. *Neuropharmacology*. **2013**; *68*, 184-194.
42. Ivanski, F., de Oliveira, V.M., de Oliveira, I.M., de Araujo Ramos, A.T., de Oliveira, Tonete, S.T., de Oliveira Hykavei, G., Bargi-Souza, P., Schiessel, D.L., Martino-Andrade, A.J., *et al*. Prepubertal acrylamide exposure causes dose-response decreases in spermatic production and functionality with modulation of genes involved in the spermatogenesis in rats. *Toxicology*. **2020**; *436*, 152428.
43. Bai, X., Hippensteel, J., Leavitt, A., Maloney, J.P., Beckham, D., Garcia, C., Li, Q., Freed, B.M., Ordway, D., Sandhaus, R.A., Chan, E.D. Hypothesis: alpha-1-antitrypsin is a promising treatment option for COVID-19. *Med Hypotheses*. **2020**; *146*, 110394.
44. Huang, Q.T., Chen, J.H., Hang, L.L., Liu, S.S., Zhong, M. Activation of PAR-1/NADPH oxidase/ROS signaling pathways is crucial for the thrombin-induced sFlt-1 production in extravillous trophoblasts: possible involvement in the pathogenesis of preeclampsia. *Cell Physiol Biochem*. **2015**; *35*, 1654-1662.
45. Yu, Q., Yang, X., Zhang, C., Zhang, X., Wang, C., Chen, L., Liu, X., Gu, Y., He, X., Hu, L., *et al*. AMPK activation by ozone therapy inhibits tissue factor-triggered intestinal ischemia and ameliorates chemotherapeutic enteritis. *FASEB J*. 132. Yu, Q., Yang, X., Zhang, C., Zhang, X., Wang, C., Chen, L., Liu, X., Gu, Y., He, X., Hu, L., *et al*. AMPK activation by ozone therapy inhibits tissue factor-triggered intestinal ischemia and ameliorates chemotherapeutic enteritis. *FASEB J*. **2020**; *34*, 13005-13021.
46. Krikun, G., Lockwood, C.J., Paidas, M.J. Tissue factor and the endometrium: from physiology to pathology. *Thromb Res*. **2009**; *124*, 393-396
47. Senol, B.K., Zulfikar, B. Clinical problems and surgical interventions in inherited factor VII deficiency. *Turk Pediatri Ars*. **2020**; *55*, 184-190.
48. Burad, J., Bhakta, P., Sharma, J.. Timely 'off-label' use of recombinant activated factor VII (NovoSeven(®)) can help in avoiding hysterectomy in intractable obstetric bleeding complicated with disseminated intravascular coagulation: A case report and review of the literature. *Indian J Anaesth*. **2012**; *56*, 69-71.
49. Gonzalez-Ramos, I.A., Mantilla-Capacho, J.M., Luna-Zaizar, H., Mundo-Ayala, J.N., Lara-Navarro, I.J., Ornelas-Ricardo, D., Gonzalez Alcazar, J.A., Evangelista-Castro, N., Jaloma-Cruz, A.R. Genetic analysis for carrier diagnosis in hemophilia A and B in the Mexican population: 25 years of experience. *Am J Med Genet C Semin Med Genet*. **2020**; *184*, 939-954.
50. Schambeck, C.M., Grossmann, R., Zonnur, S., Berger, M., Teuchert, K., Spahn, A., Walter, U. High factor VIII (FVIII) levels in venous thromboembolism: role of unbound FVIII. *Thromb Haemost*. **2004**; *92*, 42-46.
51. Lin, H.F., Maeda, N., Smithies, O., Straight, D.L., Stafford, D.W. A coagulation factor IX-deficient mouse model for human hemophilia B. *Blood*. **1997**; *90*, 3962-3966.
52. Ameri, A., Kurachi, S., Sueishi, K., Kuwahara, M., Kurachi, K. Myocardial fibrosis in mice with overexpression of human blood coagulation factor IX. *Blood*. **2003**; *101*, 1871-1873.
53. Khealani, B., Farhat, Z., Mozaffar, T. Factor XI deficiency-related spontaneous primary intraventricular hemorrhage. *South Med J*. **2000**; *93*, 1017-1018.

54. Sokol, J., Biringer, K., Skerenova, M., Stasko, J., Kubisz, P. Activity of coagulation factor XI in patients with spontaneous miscarriage: the presence of risk alleles. *J Obstet Gynaecol*. **2015**; *35*, 621-624.
55. Motegi, M., Himeno, T., Nakai-Shimoda, H., Inoue, R., Ozeki, N., Hayashi, Y., Sasajima, S., Mohiuddin, M.S., Asano-Hayami, E., Kato, M., et al. Deficiency of glucagon gene-derived peptides induces peripheral polyneuropathy in mice. *Biochem Biophys Res Commun*. **2020**; *532*, 47-53.
56. Sun, S., Liu, S., Luo, J., Chen, Z., Li, C., Loor, J.J., Cao, Y. Repeated pregnant mare serum gonadotropin-mediated oestrous synchronization alters gene expression in the ovaries and reduces reproductive performance in dairy goats. *Reprod Domest Anim*. **2019**; *54*, 873-881.
57. Jorgensen, J.O.L., Juul, A. Therapy of endocrine disease: growth hormone replacement therapy in adults: 30 years of personal clinical experience. *Eur J Endocrinol*. **2018**; *179*, R47-R56.
58. Regan, S.L.P., Knight, P.G., Yovich, J.L., Arfuso, F., Dharmarajan, A. Growth hormone during in vitro fertilization in older women modulates the density of receptors in granulosa cells, with improved pregnancy outcomes. *Fertil Steril*. **2018**; *110*, 1298-1310.
59. Kirchhoff, S., Kim, J.S., Hagendorff, A., Thonnissen, E., Kruger, O., Lamers, W.H., Willecke, K. Abnormal cardiac conduction and morphogenesis in connexin40 and connexin43 double-deficient mice. *Circ Res*. **2000**; *87*, 399-405.
60. Lanner, F., Lee, K.L., Ortega, G.C., Sohl, M., Li, X., Jin, S., Hansson, E.M., Claesson-Welsh, L., Poellinger, L., Lendahl, U., Farnebo, F. Hypoxia-induced arterial differentiation requires adrenomedullin and notch signaling. *Stem Cells Dev*. **2013**; *22*, 1360-1369.
61. Cui, Z., Agarwal, A., da Silva, B.F., Sharma, R., Sabanegh, E. Evaluation of seminal plasma proteomics and relevance of FSH in identification of nonobstructive azoospermia: a preliminary study. *Andrologia*. **2018**; *50*, e12999.
62. Dickinson, S.E., Griffin, B.A., Elmore, M.F., Kriese-Anderson, L., Elmore, J.B., Dyce, P.W., Rodning, S.P., Biase, F.H. Transcriptome profiles in peripheral white blood cells at the time of artificial insemination discriminate beef heifers with different fertility potential. *BMC Genomics*. **2018**; *19*, 129.
63. Vawter, M.P., Harvey, P.D., DeLisi, L.E. Dysregulation of X-linked gene expression in Klinefelter's syndrome and association with verbal cognition. *Am J Med Genet B Neuropsychiatr Genet*. **2007**; *144B*, 728-734.
64. Lynn, R. New evidence for dysgenic fertility for intelligence in the United States. *Soc Biol*. **1999**; *46*, 146-153.
65. Namazi, M.R. Minor thalassemia may be a risk factor for impulsiveness. *Med Hypotheses*. **2003**; *60*, 335-336.
66. Takhviji, V., Zibara, K., Azarkeivan, A., Mehrvar, N., Mehrvar, N., Mezginejad, F., Khosravi, A. Fertility and pregnancy in Iranian thalassemia patients: An update on transfusion complications. *Transfus Med.* **2020**; *30*, 352-360.
67. Elalfy, M.S., Ibrahim, A.S., Ibrahim, G.S., Hussein, H.M.A.G., Mohammed, H.G.E., Ebeid, F.S.E. Hidden brain iron content in sickle cell disease: impact on neurocognitive functions. *Eur J Pediatr*. **2021**; *180*, 2677-2686
68. Hauser, A,. Troesch, S., Steiner, T., Brocherie, F., Girard, O., Saugy, J.J., Schmitt, L., Millet, G.P., Wehrlin, J.P. Do male athletes with already high initial haemoglobin mass benefit from 'live high-train low' altitude training? *Exp Physiol*. **2018**; *103*, 68-76.
69. Li, F., Zhu, Z., Xue, M., He, W., Zhang, T., Feng, L., Lin, S.. siRNA-based breast cancer therapy by suppressing 17β-hydroxysteroid dehydrogenase type 1 in an optimized xenograft cell and molecular biology model in vivo. *Drug Des Devel Ther*. **2019**; *13*, 757-766.
70. He, W., Gauri, M., Li, T., Wang, R., Lin, S.X. Current knowledge of the multifunctional 17β-hydroxysteroid dehydrogenase type 1 (HSD17B1). *Gene*. **2016**; *588*, 54-61.
71. Sasaki, Y., Otsuka, K., Arimochi, H., Tsukumo, S.I., Yasutomo, K. Distinct roles of IL-1β and IL-18 in NLRC4-induced autoinflammation. *Front Immunol*. **2020**; *11*, 591713.
72. Olkkonen, J., Kouri, V.P., Hynninen, J., Konttinen, Y.T., Mandelin, J. Differentially expressed in chondrocytes 2 (DEC2) increases the expression of IL-1β and is abundantly present in synovial membrane in rheumatoid arthritis. *PLoS One*. **2015**; *10*, e0145279.
73. Wang, M., Wu, H., Duan, M., Yang, Y., Wang, G., Che, F., Liu, B., He, W., Li, Q., Zhang, L. SS30, a novel thioaptamer targeting CD123, inhibits the growth of acute myeloid leukemia cells. *Life Sci*. **2019**; *232*, 116663.
74. Stirewalt, D.L., Meshinchi, S., Kopecky, K.J., Fan, W., Pogosova-Agadjanyan, E.L., Engel, J.H., Cronk, M.R., Dorcy, K.S., McQuary, A.R., Hockenbery, D., *et al*. Identification of genes with abnormal expression changes in acute myeloid leukemia. *Genes Chromosomes Cancer*. **2008**; *47*, 8-20.
75. Sun, Y., Liu, S., Hu, R., Zhou, Q., Li, X. Decreased placental IL9 and IL9R in preeclampsia impair trophoblast cell proliferation, invasion, and angiogenesis. *Hypertens Pregnancy*. **2020**; *39*, 228-235.
76. Osterfeld, H., Ahrens, R., Strait, R., Finkelman, F.D., Renauld, J.C., Hogan, S.P. Differential roles for the IL-9/IL-9 receptor alpha-chain pathway in systemic and oral antigen-induced anaphylaxis. *J Allergy Clin Immunol*. **2010**; *125*, 469-476.e2.
77. Bassett, J.M., Hanson, C. Prevention of hypoinsulinemia modifies catecholamine effects in fetal sheep. *Am J Physiol Regul Integr Comp Physiol*. **2000**; *278*, R1171-R1181.
78. Landrum, M.J., Lee, J.M., Riley, G.R., Jang, W., Rubinstein, W.S., Church, D.M., Maglott, D.R. ClinVar: public archive of relationships among sequence variation and human phenotype. *Nucleic Acids Res*. **2014**; *42*, D980-D985.

79. Komura, K., Yoshikawa, Y., Shimamura, T., Chakraborty, G., Gerke, T.A., Hinohara, K., Chadalavada, K., Jeong, S.H., Armenia, J., Du, S.Y., *et al*. ATR inhibition controls aggressive prostate tumors deficient in Y-linked histone demethylase KDM5D. *J Clin Invest*. **2018**; *128*, 2979-2995.
80. Mokou, M., Klein, J., Makridakis, M., Bitsika, V., Bascands, J.L., Saulnier-Blache, J.S., Mullen, W., Sacherer, M., Zoidakis, J., Pieske, B., *et al*. Proteomics based identification of KDM5 histone demethylases associated with cardiovascular disease. *EBioMedicine*. **2019**; *41*, 91-104.
81. Chou, S.H., Chamberland, J.P., Liu, X., Matarese, G., Gao, C., Stefanakis, R., Brinkoetter, M.T., Gong, H., Arampatzi, K., Mantzoros, C.S. Leptin is an effective treatment for hypothalamic amenorrhea. *Proc Natl Acad Sci USA*. **2011**;*108*, 6585-6590.
82. Montserrat-de la Paz, S., Perez-Perez, A., Vilarino-Garcia, T., Jimenez-Cortegana, C., Muriana, F.J.G., Millan-Linares, M.C., Sanchez-Margalet, V. Nutritional modulation of leptin expression and leptin action in obesity and obesity-asociated complications. *J Nutr Biochem*. **2020**; *26*, 108561.
83. De Blasio, D., Fumagalli, S., Longhi, L., Orsini, F., Palmioli, A., Stravalaci, M., Vegliante, G., Zanier, E.R., Bernardi, A., Gobbi, M., De Simoni, M.G. Pharmacological inhibition of mannose-binding lectin ameliorates neurobehavioral dysfunction following experimental traumatic brain injury. *J Cereb Blood Flow Metab*. **2017**; *37*, 938-950.
84. Chatterjee, S.K., Saha, S., Munoz, M.N.M. Molecular pathogenesis, immunopathogenesis and novel therapeutic strategy against COVID-19. *Front Mol Biosci*. **2020**; *7*, 196.
85. Larsen, P., Yong, V.W. The expression of matrix metalloproteinase-12 by oligodendrocytes regulates their maturation and morphological differentiation. *J Neurosci*. **2004**; *24*, 7597-7603.
86. Hiden, U., Eyth, C.P., Majali-Martinez, A., Desoye, G., Tam-Amersdorfer, C., Huppertz, B., Ghaffari Tabrizi-Wizsy, N. Expression of matrix metalloproteinase 12 is highly specific for non-proliferating invasive trophoblasts in the first trimester and temporally regulated by oxygen-dependent mechanisms including HIF-1A. *Histochem Cell Biol*. **2018**; *149*, 31-42.
87. Vujovic, S., Ivovic, M., Tancic-Gajic, M., Marina, L., Ljubic, A., Dragojevic-Dikic S., Genazzani, A.R. Endometrium receptivity in premature ovarian insufficiency - how to improve fertility rate and predict diseases? *Gynecol Endocrinol*. **2018**; *34*, 1011-1015
88. Perez-Sepulveda, A., Espana-Perrot, P.P., Fernandez, X.B., Ahumada, V., Bustos, V., Arraztoa, J.A., Dobierzewska, A., Figueroa-Diesel, H., Rice, G.E., Illanes, S.E. Levels of key enzymes of methionine-homocysteine metabolism in preeclampsia. *Biomed Res Int*. **2013**; *2013*, 731962.
89. Gong, Y., Wang, L., Chippada-Venkata, U., Dai, X., Oh, W.K., Zhu, J. Constructing Bayesian networks by integrating gene expression and copy number data identifies NLGN4Y as a novel regulator of prostate cancer progression. *Oncotarget*. **2016**; *7*, 68688-68707.
90. Bhat, M.A., Sharma, J.B., Roy, K.K., Sengupta, J., Ghosh, D. Genomic evidence of Y chromosome microchimerism in the endometrium during endometriosis and in cases of infertility. *Reprod Biol Endocrinol*. **2019**; *17*, 22.
91. Shen, J., Lai, D.H., Wilson, R.A., Chen, Y.F., Wang, L.F., Yu, Z.L., Li, M.Y., He, P., Hide, G., Sun, X., et al. Nitric oxide blocks the development of the human parasite *Schistosoma japonicum. Proc Natl Acad Sci USA*. **2017**; *114*, 10214-10219.
92. Wojcik, M., Zieleniak, A., Zurawska-Klis, M., Cypryk, K., Wozniak, L.A. Increased expression of immune-related genes in leukocytes of patients with diagnosed gestational diabetes mellitus (GDM). *Exp Biol Med (Maywood)*. **2016**; *241*, 457-465.
93. Budefeld, T., Tobet, S.A., Majdic, G. Steroidogenic factor 1 and the central nervous system. *J Neuroendocrinol.* **2012**; *24*, 225-235
94. Domenice, S., Machado, A.Z., Ferreira, F.M., Ferraz-de-Souza, B., Lerario, A.M., Lin, L., Nishi, M.Y., Gomes, N.L., da Silva, T.E., Silva, R.B.,et al. Wide spectrum of NR5A1-related phenotypes in 46,XY and 46,XX individuals. *Birth Defects Res C Embryo Today*. **2016**; *108*, 309-320.
95. Fu, Y.X., Ji, J., Shan, F., Li, J., Hu, R. Human mesenchymal stem cell treatment of premature ovarian failure: new challenges and opportunities. *Stem Cell Res Ther*. **2021**; *12*, 161
96. Yilmaz, B.D., Bulun, S.E. Endometriosis and nuclear receptors. *Hum Reprod Update*. **2019**; *25*, 473-485
97. Bosch, F., Angele, M.K., Chaudry, I.H. Gender differences in trauma, shock and sepsis. *Mil Med Res*. **2018**; *5*, 35.
98. Lundin, C., Hjorth, L., Behrendtz, M., Nordgren, A., Palmqvist, L., Andersen, M.K., Biloglav, A., Forestier, E., Paulsson, K., Johansson, B. High frequency of BTG1 deletions in acute lymphoblastic leukemia in children with down syndrome. *Genes Chromosomes Cancer*. **2012**; *51*, 196-206.
99. Fujiwara, S., Yamashita, Y., Choi, Y.L., Watanabe, H., Kurashina, K., Soda, M., Enomoto, M., Hatanaka, H., Takada, S., Ozawa, K., Mano, H. Transforming activity of purinergic receptor P2Y, G protein coupled, 8 revealed by retroviral expression screening. *Leuk Lymphoma*. **2007**; *48*, 978-986.
100. Gebril, M., Hirota, Y., Aikawa, S., Fukui, Y., Kaku, T., Matsuo, M., Hirata, T., Akaeda, S., Hiraoka, T., Shimizu-Hirota, R., *et al*. Uterine epithelial progesterone receptor governs uterine receptivity through epithelial cell differentiation. *Endocrinology*. **2020**; *161*, bqaa195.
101. Wu, J.R., Zhao, Y., Zhou, X.P., Qin, X. Estrogen receptor 1 and progesterone receptor are distinct biomarkers and prognostic factors in estrogen receptor-positive breast cancer: evidence from a bioinformatic analysis. *Biomed Pharmacother*. **2020**; *121*, 109647.
102. Tian, Y., Stamova, B., Jickling, G.C., Xu, H., Liu, D., Ander, B.P., Bushnell, C., Zhan, X., Turner, R.J., Davis, R.R., *et al*. Y chromosome gene expression in the blood of male patients with ischemic stroke compared with male controls. *Gend Med*. **2012**; *9*, 68-75.e3.
103. Mithani, S.K., Smith, I.M., Califano, J.A. Use of integrative epigenetic and cytogenetic analyses to identify novel tumor-suppressor genes in malignant melanoma. *Melanoma Res*. **2011**; *21*, 298-307.

104. Gao, J., Zhang, Y., Zhang, T., Yang, Y., Yuan, C., Jia, J., Wang, Z. Responses of gonadal transcriptome and physiological analysis following exposure to 17α-ethynylestradiol in adult rare minnow Gobiocypris rarus. *Ecotoxicol Environ Saf*. **2017**; *141*, 209-215.
105. van Kempen, L.C., Redpath, M., Elchebly, M., Klein, K.O., Papadakis, A.I., Wilmott, J.S., Scolyer, R.A., Edqvist, P.H., Ponten, F., Schadendorf, D., *et al*. The protein phosphatase 2A regulatory subunit PR70 is a gonosomal melanoma tumor suppressor gene. *Sci Transl Med*. **2016**; *8*, 369ra177.
106. Dinarvand, P., Moser, K.A. Protein C deficiency. *Arch Pathol Lab Med*. **2019**; *143*, 1281-1285.
107. Lay, A.J., Liang, Z., Rosen, E.D., Castellino, F.J. Mice with a severe deficiency in protein C display prothrombotic and proinflammatory phenotypes and compromised maternal reproductive capabilities. *J Clin Invest*. **2005**; *115*, 1552-1561.
108. Ahmadi Rastegar, D., Sharifi Tabar, M., Alikhani, M., Parsamatin, P., Sahraneshin Samani, F., Sabbaghian, M., Sadighi Gilani, M.A., Mohammad Ahadi, A., Mohseni Meybodi, A., Piryaei, A., *et al*. Isoform-level gene expression profiles of human Y chromosome azoospermia factor genes and their X chromosome paralogs in the testicular tissue of non-obstructive azoospermia patients. *J Proteome Res*. **2015**; *14*, 3595-3605.
109. Yang, H., Li, Q., Zhang, L., Zhu, M., Niu, J., Xue, F., Yang, L., Qu, Q., Lao, Y., Ding, Z., *et al*. LncPRYP4-3 serves as a novel diagnostic biomarker for dissecting subtypes of metabolic associated fatty liver disease by targeting RPS4Y2. *Clin Exp Med*. **2020**; *20*, 587-600.
110. Hoffmann, S., Roeth, R., Diebold, S., Gogel, J., Hassel, D., Just, S., Rappold, G.A. Identification and tissue-specific characterization of novel SHOX-regulated genes in zebrafish highlights SOX family members among other genes. *Front Genet*. **2021**; *12*, 688808
111. German, A., Mesch, G., Hochberg, Z. People are taller in countries with better environmental conditions. *Front Endocrinol (Lausanne)*. **2020**; *11*, 106
112. Upners, E.N., Jensen, R.B., Rajpert-De Meyts, E., Dunø, M., Aksglaede, L., Juul, A. Short stature homeobox-containing gene duplications in 3.7% of girls with tall stature and normal karyotypes. *Acta Paediatr*. **2017**; *106*, 1651-1657.
113. Magann EF, Winchester MI, Carter DP, Martin JN Jr, Bass JD, Morrison JC. Factors adversely affecting pregnancy outcome in the military. Am J Perinatol. **1995**; *12*, 462-466.
114. Clemencon, B., Babot, M., Trezeguet, V. The mitochondrial ADP/ATP carrier (SLC25 family): pathological implications of its dysfunction. *Mol Aspects Med*. **2013**; *34*, 485-493.
115. Guo, X., Huang, Y., Qi, Y., Liu, Z., Ma, Y., Shao, Y., Jiang, S., Sun, Z., Ruan, Q. Human cytomegalovirus miR-UL36-5p inhibits apoptosis via downregulation of adenine nucleotide translocator 3 in cultured cells. *Arch Virol*. **2015**; *160*, 2483-2490.
116. Garratt, M., Bathgate, R., de Graaf, S.P., Brooks, R.C. Copper-zinc superoxide dismutase deficiency impairs sperm motility and in vivo fertility. *Reproduction*. **2013**; *146*, 297-304.
117. Herman, S., Lipinski, P., Ogorek, M., Starzynski, R., Grzmil, P., Bednarz, A., Lenartowicz, M. Molecular regulation of copper homeostasis in the male gonad during the process of spermatogenesis. *Int J Mol Sci*. **2020**; *21*, 9053.
118. Wang, J., Jiang, Q., Faleti, O.D., Tsang, C.M., Zhao, M., Wu, G., Tsao, S.W., Fu, M., Chen, Y., Ding, T., *et al*. Exosomal delivery of AntagomiRs targeting viral and cellular microRNAs synergistically inhibits cancer angiogenesis. *Mol Ther Nucleic Acids*. **2020**; *22*, 153-165.
119. Ning, Z., Williams, J.M., Kumari, R., Baranov, P.V., Moore, T. Opposite expression patterns of spry3 and p75NTR in cerebellar vermis suggest a male-specific mechanism of autism pathogenesis. *Front Psychiatry*. **2019**; *10*, 416.
120. Caron, K.M., Soo, S.C., Wetsel, W.C., Stocco, D.M., Clark, B.J., Parker, K.L. Targeted disruption of the mouse gene encoding steroidogenic acute regulatory protein provides insights into congenital lipoid adrenal hyperplasia. *Proc Natl Acad Sci USA*. **1997**; *94*, 11540-11545.
121. Horvath, A., Mathyakina, L., Vong, Q., Baxendale, V., Pang, A.L., Chan, W.Y., Stratakis, C.A. Serial analysis of gene expression in adrenocortical hyperplasia caused by a germline PRKAR1A mutation. *J Clin Endocrinol Metab*. **2006**; *91*, 584-596.
122. Meyfour, A., Ansari, H., Pahlavan, S., Mirshahvaladi, S., Rezaei-Tavirani, M., Gourabi, H., Baharvand, H., Salekdeh, G.H. Y chromosome missing protein, TBL1Y, may play an important role in cardiac differentiation. *J Proteome Res*. **2017**; *16*, 4391-4402.
123. Sood, R., Sholl, L., Isermann, B., Zogg, M., Coughlin, S.R., Weiler, H. Maternal Par4 and platelets contribute to defective placenta formation in mouse embryos lacking thrombomodulin. *Blood*. **2008**; *112*, 585-591.
124. Asai, Y., Yamamoto, T., Kito, D., Ichikawa, K., Abe, Y. Factors influencing the effectiveness of recombinant human soluble thrombomodulin on disseminated intravascular coagulation: a retrospective study. *J Pharm Health Care Sci*. **2020**; *6*, 26.
125. Dasari, V.K., Goharderakhshan, R.Z., Perinchery, G., Li, L.C., Tanaka, Y., Alonzo, J., Dahiya, R. Expression analysis of Y chromosome genes in human prostate cancer. *J Urol*. **2001**; *165*, 1335-1341.
126. Wong, H.Y., Wang, G.M., Croessmann, S., Zabransky, D.J., Chu, D., Garay, J.P., Cidado, J., Cochran, R.L., Beaver, J.A., Aggarwal, A., *et al*. TMSB4Y is a candidate tumor suppressor on the Y chromosome and is deleted in male breast cancer. *Oncotarget*. **2015**; *6*, 44927-44940.
127. Wang, X., Dong, C., Sun, L., Zhu, L., Sun, C., Ma, R., Ning, K., Lu, B., Zhang, J., Xu, J. Quantitative proteomic analysis of age-related subventricular zone proteins associated with neurodegenerative disease. *Sci Rep*. **2016**; *6*, 37443..

128. Yu, W.L., Yu, G., Dong, H., Chen, K., Xie, J., Yu, H., Ji, Y., Yang, G.S., Li, A.J., Cong, W.M, Jin, G.Z. Proteomics analysis identified TPI1 as a novel biomarker for predicting recurrence of intrahepatic cholangiocarcinoma. *J Gastroenterol*. **2020**; *55*, 1171-1182.
129. Halder, A., Kumar, P., Jain, M., Iyer, V.K. Copy number variations in testicular maturation arrest. *Andrology*. **2017**; *5*, 460-472.
130. Gegenschatz-Schmid, K., Verkauskas, G., Stadler, M.B., Hadziselimovic, F. Genes located in Y-chromosomal regions important for male fertility show altered transcript levels in cryptorchidism and respond to curative hormone treatment. *Basic Clin Androl*. **2019**; *29*, 8.
131. Jedidi, I., Ouchari, M., Yin, Q. Sex chromosomes-linked single-gene disorders involved in human infertility. *Eur J Med Genet*. **2019**; *62*, 103560
132. Heidecker, B., Lamirault, G., Kasper, E.K., Wittstein, I.S., Champion, H.C., Breton, E., Russell, S.D., Hall, J., Kittleson, M.M., Baughman, K.L., Hare, J.M. The gene expression profile of patients with new-onset heart failure reveals important gender-specific differences. *Eur Heart J*. **2010**; *31*, 1188-1196.
133. Shpargel, K.B., Sengoku, T., Yokoyama, S., Magnuson, T. UTX and UTY demonstrate histone demethylase-independent function in mouse embryonic development. *PLoS Genet*. **2012**; *8*, e1002964.
134. Pottmeier, P., Doszyn, O., Peuckert, C., Jazin, E. Increased expression of Y-encoded demethylases during differentiation of human male neural stem cells. *Stem Cells Dev*. **2020**; *29*, 1497-1509.
135. Zhu, L., Dong, L., Feng, M., Yang, F., Jiang, W., Huang, Z., Liu, F., Wang, L., Wang, G., Li, Q. Profiles of autophagy-related genes in esophageal adenocarcinoma. *BMC Cancer*. **2020**; *20*, 943.
136. Tannour-Louet, M., Han, S., Louet, J.F., Zhang, B., Romero, K., Addai, J., Sahin, A., Cheung, S.W., Lamb, D.J. Increased gene copy number of VAMP7 disrupts human male urogenital development through altered estrogen action. *Nat Med*. **2014**; *20*, 715-724.
137. Radko, S., Koleva, M., James, K.M., Jung, R., Mymryk, J.S., Pelka, P. Adenovirus E1A targets the DREF nuclear factor to regulate virus gene expression, DNA replication, and growth. *J Virol*. **2014**; *88*, 13469-13481.
138. Xi, J.F., Wang, X.Z., Zhang, Y.S., Jia, B., Li, C.C., Wang, X.H., Ying, R.W. Sex control by Zfy siRNA in the dairy cattle. *Anim Reprod Sci*. **2019**; *200*, 1-6.
139. Jan, S.Z., Jongejan, A., Korver, C.M., van Daalen, S.K.M., van Pelt, A.M.M., Repping, S., Hamer, G. Distinct prophase arrest mechanisms in human male meiosis. *Development*. **2018**; *145*, dev160614.

© 2021 by the authors. Submitted for possible open access publication under the terms and conditions of the Creative Commons Attribution (CC BY) license (http://creativecommons.org/licenses/by/4.0/).
